# Detection of phenotype‐specific therapeutic vulnerabilities in breast cells using a CRISPR loss‐of‐function screen

**DOI:** 10.1002/1878-0261.12951

**Published:** 2021-05-01

**Authors:** Anna Barkovskaya, Craig M. Goodwin, Kotryna Seip, Bylgja Hilmarsdottir, Solveig Pettersen, Clint Stalnecker, Olav Engebraaten, Eirikur Briem, Channing J. Der, Siver A. Moestue, Thorarinn Gudjonsson, Gunhild M. Mælandsmo, Lina Prasmickaite

**Affiliations:** ^1^ Department of Tumor Biology Institute for Cancer Research Oslo University Hospital The Norwegian Radium Hospital Oslo Norway; ^2^ Department of Circulation and Medical Imaging Norwegian University of Science and Technology Trondheim Norway; ^3^ Lineberger Comprehensive Cancer Center University of North Carolina at Chapel Hill NC USA; ^4^ Biomedical Center School of Health Sciences University of Iceland Reykjavik Iceland; ^5^ Department of Pathology Landspitali University Hospital Reykjavik Iceland; ^6^ Institute of Clinical Medicine Faculty of Medicine University of Oslo Norway; ^7^ Department of Oncology Oslo University Hospital Norway; ^8^ Department of Genetics and Molecular Medicine Landspitali University Hospital Reykjavik Iceland; ^9^ Department of Health Sciences Nord University Bodø Norway; ^10^ Department of Laboratory Hematology Landspitali University Hospital Reykjavik Iceland; ^11^ Faculty of Health Sciences Institute of Medical Biology The Arctic University of Norway – University of Tromsø Norway; ^12^ Present address: Department of Surgery Center for Bioengineering & Tissue Regeneration University of California San Francisco CA USA

**Keywords:** actionable targets, CRISPR knockout screen, epithelial–mesenchymal transition, phenotype plasticity, therapeutic vulnerabilities, triple‐negative breast cancer

## Abstract

Cellular phenotype plasticity between the epithelial and mesenchymal states has been linked to metastasis and heterogeneous responses to cancer therapy, and remains a challenge for the treatment of triple‐negative breast cancer (TNBC). Here, we used isogenic human breast epithelial cell lines, D492 and D492M, representing the epithelial and mesenchymal phenotypes, respectively. We employed a CRISPR‐Cas9 loss‐of‐function screen targeting a 2240‐gene ‘druggable genome’ to identify phenotype‐specific vulnerabilities. Cells with the epithelial phenotype were more vulnerable to the loss of genes related to EGFR‐RAS‐MAPK signaling, while the mesenchymal‐like cells had increased sensitivity to knockout of G_2_‐M cell cycle regulators. Furthermore, we discovered knockouts that sensitize to the mTOR inhibitor everolimus and the chemotherapeutic drug fluorouracil in a phenotype‐specific manner. Specifically, loss of EGFR and fatty acid synthase (FASN) increased the effectiveness of the drugs in the epithelial and mesenchymal phenotypes, respectively. These phenotype‐associated genetic vulnerabilities were confirmed using targeted inhibitors of EGFR (gefitinib), G_2_‐M transition (STLC), and FASN (Fasnall). In conclusion, a CRISPR‐Cas9 loss‐of‐function screen enables the identification of phenotype‐specific genetic vulnerabilities that can pinpoint actionable targets and promising therapeutic combinations.

## Graphical abstract

Phenotypic heterogeneity hampers anticancer treatment efficacy. Here, we applied a CRISPR‐Cas9 loss‐of‐function screen of a ‘druggable genome’ in isogenic breast cell lines displaying opposing EMT phenotypes. We identified phenotype‐specific genetic vulnerabilities (alone or under ‘therapy pressure’) that represent actionable targets in distinct phenotypes: EGFR‐MAPK signaling in the epithelial phenotype, and G_2_‐M transition and FASN in the mesenchymal phenotype cells.

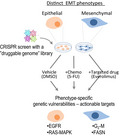

**Abbreviations**
5‐FU: fluorouracil
BC: breast cancer
CRISPR: clustered regularly interspaced short palindromic repeats
CTG: CellTiter‐Glo
EG5: kinesin 5
EGFR: epidermal growth factor receptor
EMT: epithelial–mesenchymal transition
FASN: fatty acid synthase
mTOR: mammalian target of rapamycin
RPPA: reverse‐phase protein array
RSA: redundant siRNA analysis
sgRNA: single‐guide RNA
STLC: S‐trityl‐l‐cysteine inhibitor
TGF‐β: transforming growth factor‐beta
TNBC: triple‐negative breast cancer

## 1 Introduction

Despite the substantial improvements in therapy over the last few decades, high‐risk and metastatic breast cancer (BC) continues to be a demanding clinical challenge, causing the death of over 600 000 women globally each year [1]. Triple‐negative breast cancer (TNBC; estrogen and progesterone receptors and HER2‐negative) has the worst clinical outcome and lacks effective treatment options. The failure of BC management is caused, in part, by the phenotypic plasticity of the cancer cells that contributes to metastasis and heterogeneous responses to therapy facilitating treatment resistance [2, 3]. Phenotype plasticity, encompassing both epithelial–mesenchymal transition (EMT) and the reverse process, describes the cells' ability to interconvert between phenotypic states along the EMT spectrum [4]. The epithelial phenotype characterizes polarized cells with cytokeratin‐based cytoskeleton that are interconnected through E‐cadherin‐based junctions. The transition to a mesenchymal phenotype includes the loss of polarity, the shift to a vimentin‐based cytoskeleton, reduced cell–cell adhesion associated with an E‐cadherin to N‐cadherin switch, and increased motility and invasiveness [5]. EMT features, high phenotypic heterogeneity, and plasticity are frequently observed in TNBC [3, 6]. Cells carrying a mesenchymal phenotype are considered to be more resistant to conventional therapies [7]. In addition, therapy‐induced switching between phenotypic states has been linked to acquired resistance [3, 8]. Since distinct phenotypic states can show different sensitivity to drugs, and phenotype switching can confer tolerance to the applied treatment, a combination of phenotype‐specific drugs could be beneficial. In line with this, computational simulations predict that combination therapy regimens that frequently alternate between epithelial‐ and mesenchymal‐specific treatments could have enhanced benefit [9]. It is therefore important to identify genes or pathways that represent phenotype‐associated vulnerabilities and could be exploited as actionable targets in a phenotype‐specific manner.

Here, we set out to identify phenotype‐specific vulnerability nodes in the epithelial and mesenchymal phenotype cells. We made use of an isogenic pair of human breast epithelial cell lines, D492 and D492M [10, 11, 12]. Similar to other known models of EMT that consist of isogenic pairs of breast cell lines, such as HMLE and PMC42 [13, 14], D492 and D492M cells display distinct, epithelial and mesenchymal phenotypes, respectively. Using the D492‐D492M cell system, we performed a clustered regularly interspaced short palindrome repeat (CRISPR) loss‐of‐function screen employing a 2240‐gene ‘druggable genome’ library covering targetable proteins or proteins in targetable pathways. We sought to discover genes and pathways that are selectively critical alone for either of the phenotypes, or selectively critical when lost in combination with drug treatment. For the latter, we combined a CRISPR‐Cas9 loss‐of‐function screen with compounds that are clinically used for the treatment of BC. We selected fluorouracil (5‐FU), a conventional cytotoxic chemotherapeutic agent, and everolimus, a targeted inhibitor of the mammalian target of rapamycin (mTOR) serine/threonine kinase, as two mechanistically distinct types of therapies.

5‐FU inhibits thymidylate synthase, leading to the disruption of DNA and RNA synthesis and repair, which results in cell death [15]. 5‐FU and its prodrug capecitabine are recommended for patients with locally advanced or metastatic BC and as a salvage therapy in nonresponding BC undergoing chemotherapy before surgery [16, 17]. A relatively high tolerance for 5‐FU‐based drugs makes them suitable for combination treatments. Multiple clinical trials testing capecitabine together with other forms of chemotherapy have demonstrated a moderate increase in disease‐free survival in BC [18, 19].

Everolimus targets mTOR, one of the major nodes of the oncogenic PI3K‐AKT‐mTOR signaling pathway [20], which is often activated in BC, also in TNBC [21]. It has been reported that the activation of the PI3K‐AKT‐mTOR signaling is associated with EMT in both normal immortalized cells and in a range of cancer cell lines [22, 23, 24]. Everolimus is approved for the treatment of estrogen receptor‐positive BC in combination with hormone therapy, where it significantly improves progression‐free survival [25]. In TNBC, clinical trials of combination treatments with everolimus have not revealed clear benefit ([26] and reviewed in Ref. [21]). Preclinical studies *in vivo* attempted to identify which subtype of TNBC may benefit from mTOR inhibitors. One study found that everolimus had favorable activity against basal‐like subtype [27], but in another study, response to everolimus was not restricted to a specific TNBC subtype [28].

Here, we aimed to identify selective genetic dependencies impacting the fitness of the epithelial or mesenchymal phenotype breast cells, and to discover actionable targets that improve the efficacy of either 5‐FU or everolimus in a phenotype‐specific manner.

## 2 Materials and methods

### 2.1 Cell culture

D492 and D492M cell lines were established as described previously by Gudjonsson *et al*. [10] and Sigurdsson *et al*. [11], respectively. Briefly, a breast epithelial progenitor cell line D492 was generated by isolating the MUC1^−^/EpCAM^+^ suprabasal cells from normal primary tissue and immortalizing them with HPV16‐E6/E7 oncogenes [10]. Such immortalization leads to inactivation of the p53 and Rb proteins (often inactivated in BC), but does not compromise essential functions of cell differentiation/polarization [29]. Therefore, the E6/E7 immortalized cell line has relevance for studies on breast morphogenesis and BC, as discussed previously [10, 29]. D492 cells are progenitor cells that can generate both luminal and myoepithelial cells in culture and express several markers of both lineages [10, 12]. As accumulating evidence links progenitor cells in the mammary gland to BC (reviewed in Ref. [30]), D492 is an interesting cell model for BC research.

When cocultured with breast endothelial cells in Matrigel, the D492 cells spontaneously undergo EMT, forming spindle‐like colonies. The cells from a spindle‐like colony were isolated and clonally expanded giving rise to the daughter cell line, D492M, which displays a stable mesenchymal phenotype [11].

D492 and D492M cells were grown in H14 medium as previously described [10, 11]. Briefly, serum‐free DMEM‐F12 medium [Thermo Fisher Scientific (TFS), Waltham, MA, USA, 31331138] was supplemented with penicillin and streptomycin (TFS, 15070‐063), 250 ng·mL^−1^ insulin (Sigma, St.Louis, MO, USA, I1882), 10 ng·mL^−1^ EGF (PeproTech, Cranbury, NJ, USA, AF‐100‐15), 10 µg·mL^−1^ transferrin (Sigma, T1147), 2.6 ng·mL^−1^ NaSel (BD Biosciences, San Jose, CA, USA, 534201), 10^−10^ m estradiol (Sigma, E2758), 500 ng·mL^−1^ hydrocortisone (Sigma, H0888), and 0.15 IU prolactin (Sigma, L6520). For passaging, cells were detached using 0.25% trypsin‐EDTA (TFS, 25200056), and trypsin was eventually inhibited by adding soybean trypsin inhibitor (Sigma, T6522).

### 2.2 Drugs and reagents

5‐FU (50 mg·mL^−1^, Accord Healthcare, London, UK) was purchased from the pharmacy at the Radium Hospital, Oslo University Hospital (Oslo, Norway), everolimus (SML2282) and Fasnall (SML1815) were purchased from Sigma‐Aldrich, gefitinib (Iressa) was purchased from Astra Zeneca, and *S*‐trityl‐l‐cysteine inhibitor STLC (2191) was purchased from Tocris Biotechne (Abingdon, UK). SCH772984 (S7101) was purchased from Selleck Chemicals (Houston, TX, USA).

### 2.3 Protein expression analysis

The level of phenotype‐specific proteins was measured using a simple western immunoassay on a Peggy Sue™ instrument (ProteinSimple, San Jose, CA, USA). The cells were lysed in lysis buffer (1% Triton X‐100, 50 mm, 150 mm NaCl, 1.5 mm MgCl_2_, 1 mm EGTA, 100 mm NaF, 10 mm Na pyrophosphate, 1 mm Na_3_VO_4_, 10% glycerol), supplemented with protease and phosphatase inhibitors (Roche Applied Science, Mannheim, Germany), followed by ultrasonication. The concentration was adjusted to 0.8 μg·μL^−1^. Protein separation was performed using a 12–230 kDa separation master kit in accordance with the manufacturer's protocol. Primary antibody incubation time was adjusted to 60 min, while all the other settings were kept on default. The compass software (ProteinSimple, version 2.7.1) was used to program the experimental setup and to collect and analyze the data. The following antibodies were used: anti‐β‐actin (A5316, Sigma) 1 : 100, anti‐E‐cadherin (MAB1838, R&D Systems, Minneapolis, MN, USA) 1 : 50, anti‐Cytokeratin 14 (NCL‐L‐LL002, Leica Biosystems, Buffalo Grove, IL, USA) 1 : 40, anti‐cytokeratin 17 (M7046, Dako, Glostrup, Danmark) 1 : 40, anti‐N‐cadherin (610920, BD Transduction Laboratories, San Jose, CA, USA) 1 : 50, and anti‐vimentin (550513, BD PharMingen, San Diego, CA, USA) 1 : 50.

For high‐throughput analysis (302 proteins), the cell lysates were analyzed by reverse‐phase protein array (RPPA) at MD Anderson RPPA core facility (Houston, TX, USA). In brief, serial dilutions of the lysates were arrayed on nitrocellulose‐coated slides. A primary antibody (specified at the core facility's home page; available upon request) was added to probe each slide, followed by a biotin‐conjugated secondary antibody. A chromogenic reaction was used to detect the signal. After scanning the slides, spot intensities were determined by analysis with the microvigene software (VigeneTech, Carlisle, MA, USA). Each dilution series was fitted with a logistic model (‘Supercurve Fitting’) to obtain a dilution curve in log2 scale. The data in log2 scale were transformed to linear and median‐centered values for each protein. The signal for the protein of interest was normalized to the signal of histone 3 in each sample (the levels of histone 3 were similar between the samples).

### 2.4 Cell viability and proliferation assays

For experiments in adherent two‐dimensional (2D) cell cultures, 4000 cells were seeded in 96‐well plates. D492 cell growth was monitored using the Incucyte^®^ instrument (Essen BioScience, Hertfordshire, UK), which tracks cell confluence over time. The viability was evaluated by measuring cell metabolic activity by CellTiter‐Glo^®^ (CTG) assay (Promega, Madison, WI, USA), adding CTG directly to the wells (1 : 1). After 10 min, bioluminescence was measured by Victor™ X3 Multiplate Reader (Perkin Elmer, Waltham, MA, USA).

For three‐dimensional (3D) cultures in Matrigel, 2500 cells were suspended in 50 µL Matrigel (Corning, Corning, NY, USA, 354230) and plated into the 96‐well plates (Corning, 10517742). After 30 min at 37 °C, 100 µL of EGM5 medium (Lonza, Basel, Switzerland, CC‐3162, containing 50 IU·mL^−1^ penicillin, 50 µg·mL^−1^ streptomycin, hydrocortisone, FGF, EGF, VEGF, R3‐IGF‐1, ascorbic acid, heparin, and supplemented with 5% FBS) was added on top of the Matrigel. The next day, the medium was replaced with H14, and a day after, the drugs were added. The medium with/without drugs was replaced every 3 days, and after 8 days, cell viability was determined using the CTG assay, when the medium was removed and CTG was added directly on top of Matrigel (50 µL/well). The plates were kept on a vigorous shaker for 1 h prior to the measurement of bioluminescence as described above. In addition, the 3D colonies were stained with the tetrazolium dye (Sigma, M5655) for visualization. For that, the medium was removed, 50 µL of 0.4 mg·mL^−1^ dye was added, and after 1 h at 37 °C the cultures were imaged using the GelCount instrument (Oxford Optronix, Abingdon, UK).

### 2.5 Immunofluorescence

For staining of 3D colonies, the D492 and D492M cells were seeded in drops of 50 µL Matrigel in cytoslides (Ibidi, Gräfelfing, Germany) and incubated for 8 days in H14 medium. After fixation with 4% paraformaldehyde (PFA, Chemi‐Teknik, Oslo, Norway) for 10 min, the colonies were stained with Alexa Fluor™ 546 phalloidin (Molecular Probes, Eugene, OR, USA, #A22283, 1 : 40) to label F‐actin. After overnight incubation at 4 °C, the stained cultures were washed and submerged in PBS before imaging.

For staining with anti‐EGFR, the 2D cell cultures on cytoslides were fixed in 4% PFA. The unspecific binding was blocked with 10% horse serum for 1 h. The cytoslides were stained with rabbit anti‐EGFR XP^®^ (Cell Signaling, Danvers, MA, USA, #4267) diluted 1 : 50 in immunofluorescence buffer (PBS with 0.1% BSA, 0.2% Triton X‐100, 0.05% Tween‐20) supplemented with 1% horse serum over night at 4 °C followed by staining with donkey anti‐rabbit DyLight 550 (Thermo Fisher, Waltham, MA, USA) for 1 h. The cytoslides were mounted with ProLong™ Gold Antifade Reagent with DAPI (Invitrogen, Carlsbad, CA, USA, #P‐36931) before imaging.

The imaging was performed using laser scanning confocal microscope (LSM710; Carl Zeiss, Oberkochen, Germany), equipped with Plan‐Apochromat ×63/1.4 Oil DICIII objective for 2D images and EC Plan‐Neofluar ×10 objective for 3D cultures. Image processing and visualization were performed by using the zen light 2011 software (Oberkochen, Germany).

### 2.6 Flow cytometry

D492 and D492M cells were collected on ice, fixed for 10 min in 1.6% PFA, and permeabilized in 100% ice‐cold methanol. Control and drug‐treated samples were stained at room temperature for 30 min with varying concentrations of Pacific Orange dye (TFS) ranging from 0 to 2 ng·μL^−1^ for barcoding of the samples. Subsequently, the samples were washed and pooled for staining with anti‐phospho‐S6 Alexa 647 (Cell Signaling Technology, #4851 dilution 1 : 200) for 30 min at room temperature.

For cell cycle analysis, the cells were stained with 20 µg·mL^−1^ Hoechst 33342 (Life Technology, Carlsbad, CA, USA, #H3570) for 60 min at 37 °C. The samples were analyzed on an LSR II flow cytometer (BD Bioscience). BD FACSDiva™ and flowjo software (FlowJo, Ashland, OR, USA) were used to collect and analyze the data.

### 2.7 Preparation of the CRISPR library and lentiviral production

The ‘druggable genome’ CRISPR knockout library (described previously [31, 32]) was generously provided by K. C. Wood (Duke University, NC, USA). This pooled library consists of lentiviral plasmids (LentiCRISPR V2), each encoding a single‐guide RNA (sgRNA), the CRISPR‐associated nuclease (Cas9), and a gene conferring resistance to puromycin. sgRNAs facilitated targeted introduction of double‐strand DNA breaks by Cas9 into the coding regions of the genes of interest. Five unique sgRNA constructs were chosen targeting 2240 genes encoding for all members of the protein kinome, chromatin modifiers, regulators of the DNA damage response, targets of FDA‐approved drugs for any indication, proteins mutated in cancer, and components of pathways dysregulated in tumorigenesis, tumor maintenance, and drug resistance.

The library was cloned and prepared as previously described [33]. Library DNA stock was amplified in Lucigen 10G ELITE Electrocompetent bacteria and cultured on agar plates to achieve at least one million distinct colonies. The colonies were collected, and the DNA was isolated using a Midi Kit (Qiagen, Hilden, Germany).

The lentivirus carrying the library was generated in HEK293T cells. HEK293T cells were seeded at 5 × 10⁶ in a T75 culture flask in DMEM supplemented with 10% FBS. The next day, a transfection mix consisting of 9 µg packaging plasmid Pax2, 3 µg envelope plasmid Pmd2G, and 15 µg CRISPR library was prepared in 200 µL Opti‐MEM (Gibco, Paisley, UK). In a separate tube, FuGENE transfection reagent (Promega, Madison, WI, USA) was added to 1 mL of Opti‐MEM at 1 : 13 dilution. After a 30‐min incubation, the contents of both tubes were combined and added to the HEK293T cells in serum‐containing medium. Following an overnight incubation, the medium was replaced with DMEM containing 20% FBS. Two days later, the medium containing lentivirus was collected, centrifuged at 200 ***g*** for 5 min, and filtered through a 0.45‐µm filter. The virus stock [after detection of multiplicity of infection (MOI) using standard protocols] was stored at −80 °C until further use.

### 2.8 CRISPR loss‐of‐function screen

The screen was conducted by first plating 5 × 10^5^ D492 and D492M cells per well into 25 6‐well plates. The following day, virus at MOI of 0.2 and polybrene (8 µg·mL^−1^) were added to the cells. Plates were centrifuged at 800 ***g*** for 1 h and incubated overnight at 37 °C. One day after lentiviral transduction, the transduction medium was replaced with fresh medium containing puromycin at 2 µg·mL^−1^. One day later, the surviving cells were trypsinized and re‐plated on 500‐cm^2^ culture plates. Simultaneously, an initial sample (T0) was flash‐frozen in liquid N_2_. Cells were propagated in the presence of puromycin for 7 days to allow complete DNA alternation. At that point, cells were split into two replicate treatment groups—1 µm 5‐FU or 5 nm everolimus drug or DMSO control—with 12 × 10^6^ cells per condition. Simultaneously, a (T7) sample was flash‐frozen. The cells were continuously cultured in the presence of 5‐FU, everolimus, or DMSO vehicle for an additional two or four weeks before the cells were collected and flash‐frozen. Cells were split as necessary to maintain sub‐90% confluency and no fewer than 12 × 10^6^ cells to maintain sequence diversity.

Genomic DNA was isolated and prepared for sequencing as previously described [33]. Briefly, at least 10 million cells were processed to extract total genomic DNA (Qiagen Blood and Tissue DNA Extraction Kit). The samples were cleaned by ethanol precipitation, and then run in two subsequent PCR steps as described in Ref. [33], with the PCR step 1 consisting of eight parallel 100 µL reactions of 5 µg DNA each, and step 2 adding a condition‐specific barcode sequence for subsequent deconvolution. The entire PCR product from the second reaction step was run through a 2% agarose gel and extracted using a Gel Extraction Kit (Qiagen), ethanol precipitated, and its concentration tested with a Qbit (Life Technologies). Sequencing was performed on an Illumina NextSeq 500 (San Diego, CA, USA) with 75bp, single‐end reads. A final concentration of 3 pmol DNA was loaded with a PhiX spike of greater than 15% to enhance signal complexity.

### 2.9 Data analyses

Sequence counts were deconvolved to separate treatment groups from total sequence reads, and then, sgRNA reads were counted for each construct as previously described [33]. To analyze samples using RSA (redundant siRNA analysis), the number of counts for each construct was normalized to the total number of counts in the same sample and averaged across both replicates. By comparing the number of cells containing each sgRNA in the D492 and D492M cells, we determined the relative effect on viability that each sgRNA had in one cell line with respect to the other. By comparing the number of cells containing each sgRNA in the drug‐treated *versus* nontreated cells, we determined the relative effect on viability that each sgRNA had in combination with the treatment. Genes were ranked and assigned significance using RSA [34].

For screen analysis using MAGeCK (model‐based analysis of genome‐wide CRISPR‐Cas9 knockout), we ran MAGeCK using MLE (maximum‐likelihood estimation) with treatment‐deconvolved FASTQ files, normalized to control (nontargeting) sgRNA [35, 36]. Screen and sequence quality analysis was provided by MAGeCK‐VISPR.

Heat maps were designed using the R plugin *pheatmap*. Qiagen Ingenuity Pathway Analysis (IPA) was used to determine pathway enrichment in the hit lists. The hits were used as input for STRING visualization [37]. A network of direct protein—protein interactions was built based on the highest confidence (0.9) evidence, and functional enrichment (KEGG/Reactome pathways) was determined using the STRING web tool (http://www.string‐db.org).

## 3 Results

### 3.1 D492 and D492M cells carry distinct phenotypes

**Fig. 1:**
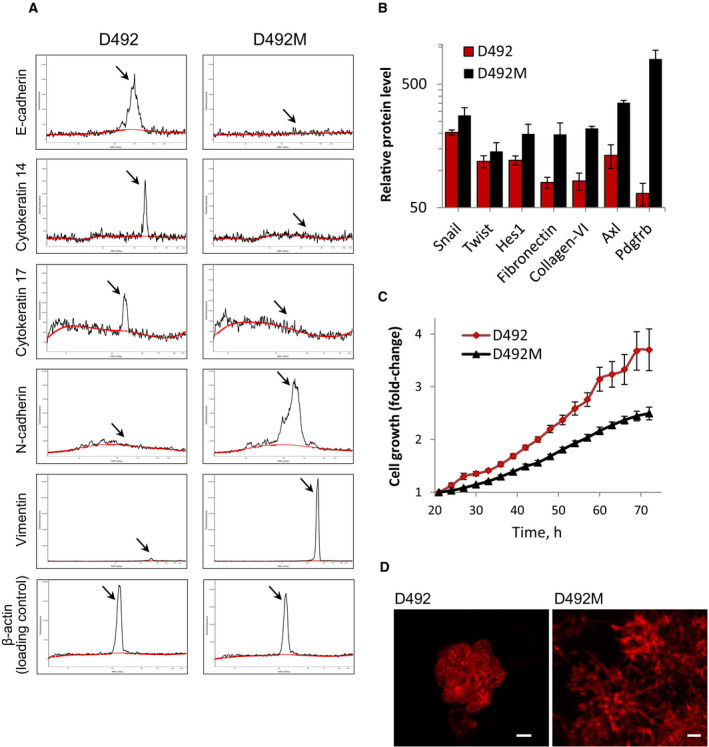
D492 and D492M cells carry an epithelial and a mesenchymal phenotype, respectively. (A, B) Relative protein levels of phenotype‐specific markers in D492 and D492M cells as measured by simple western immunoassay (A; representative electropherograms, where the *x*‐axis shows the protein size (kDa), and the *y*‐axis indicates signal intensity, reflecting the amount of the protein), and the RPPA (B; average ± SD from three technical replicates). (C) Cell growth shown as increase in confluence (*y*‐axis) during time after seeding (*x*‐axis) tracked by the Incucyte (average ± SEM, *n* ≥ 3). (D) D492 and D492M cell colonies formed during 8‐day growth in 3D Matrigel and stained with phalloidin (red) for labeling F‐actin; scale bar, 50 µm.

To validate that D492 and D492M cells retain their previously described characteristics reflecting epithelial and mesenchymal phenotypes, respectively, we first analyzed the expression of known markers. In agreement with previous studies [10, 11, 12], we determined that D492 cells express high levels of epithelial markers such as E‐cadherin and cytokeratins, while the D492M cells lost the expression of these proteins and gained expression of mesenchymal markers, including N‐cadherin, vimentin, and several others (Fig. 1A,B). When testing cell proliferation in 2D cultures, we observed faster growth of D492 cells compared with D492M (Fig. 1C), which is a common difference for their respective phenotypes. When cultured in 3D Matrigel, D492 forms branching lobular‐like structure characteristic for epithelial cells, whereas D492M shows invasive behavior typical for mesenchymal‐like cells (Fig. 1D). Altogether, this confirms that D492 cells represent the epithelial faster‐growing phenotype, while D492M cells display the mesenchymal invasive phenotype.

### 3.2 Determining sensitivity of D492 and D492M cells to 5‐FU and everolimus

**Fig. 2:**
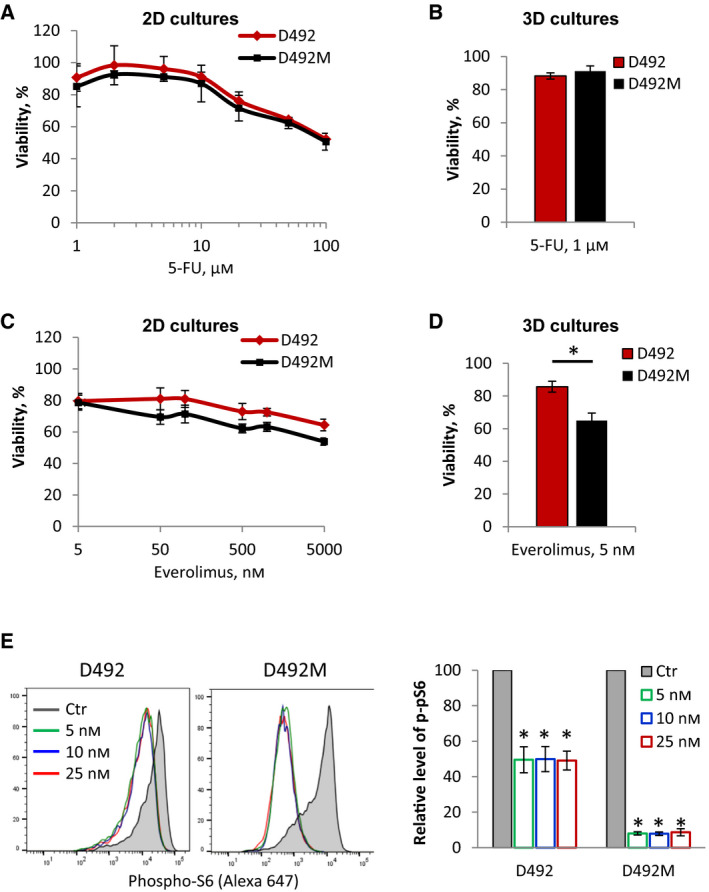
Sensitivity of D492 and D492M cells to 5‐FU and everolimus. (A) D492 and D492M cells growing in 2D were treated with increasing concentration of 5‐FU for 3 days before the cell survival was measured by the CTG assay; average ± SD (*n* = 2). (B) Viability of D492 and D492M cells in 3D Matrigel treated with 1 µm 5‐FU for 8 days before the cell survival was measured by the CTG assay; average ± SEM (*n* ≥ 8). (C) D492 and D492M cells growing in 2D were treated with increasing concentration of everolimus for 3 days before the cell survival was measured by the CTG assay; average ± SEM (*n* ≥ 4). (D) Viability of the D492 and D492M cells in 3D Matrigel treated with 5 nm everolimus for 8 days before the cell survival was measured by the CTG assay; average ± SEM (*n* ≥ 9). (E) Reduction in the level of phospho‐S6 (as measured by flow cytometry) in D492 and D492M cells in response to 2 days of treatment with indicated concentrations of everolimus; left: representative histograms; right: relative level of phospho‐S6 quantified by normalizing the levels in the treated samples to the levels in the nontreated respective controls (set to 100); average ± SEM (*n* = 3); *P ≤ 0.05, unpaired *t*‐test.

Because our CRISPR screen in the presence of ‘therapy pressure’ would require continuous treatment with the drug for up to four weeks, we set out to determine sublethal drug doses. Dose–response studies in 2D cultures revealed that a 3‐day treatment with 5‐FU up to 10 µm reduced cell viability by approximately 10% in both cell lines (Fig. 2A). Tracking cell density over a 6‐day period confirmed that a dose of 1 µm 5‐FU reduces cell proliferation in a statistically significant manner (Fig. S1A). This dose caused approximately 10% reduction in viability when the cells were cultured in Matrigel in 3D and treated for 8 days (Fig. 2B). Based on these data, a 1 µm dose of 5‐FU was selected to be used in the CRISPR screen.

A 3‐day treatment with everolimus up to 100 nm reduced cell viability in 2D cultures by 20–30% (Fig. 2C). D492M cells seemed to be slightly more sensitive than D492, although the difference was not statistically significant. However, an 8‐day treatment with 5 nm everolimus in 3D cultures in Matrigel confirmed a higher sensitivity of D492M cells, whose viability was reduced by approximately 35% compared with 15% in D492 cells (Fig. 2D). Further, 5 nm everolimus suppressed the mTOR signaling pathway as detected by decreased phosphorylation of its downstream target, S6 ribosomal protein, and no further decrease in phospho‐S6 levels was observed with higher doses of everolimus (Fig. 2E). The effect of everolimus on phospho‐S6 was stronger in D492M cells. The higher sensitivity of D492M cells might be attributed to their dependence on mTOR signaling. Indeed, the protein levels of mTOR and its downstream targets were higher in D492M cells compared with D492 (Fig. S1B), which is in line with the previous reports that link PI3K‐AKT‐mTOR signaling to EMT [23, 38, 39]. Based on these data, we chose to use 5 nm of everolimus for both cell lines in the CRISPR screen.

### 3.3 CRISPR screen uncovers genetic vulnerabilities specific for D492 or D492M cells

**Fig. 3:**
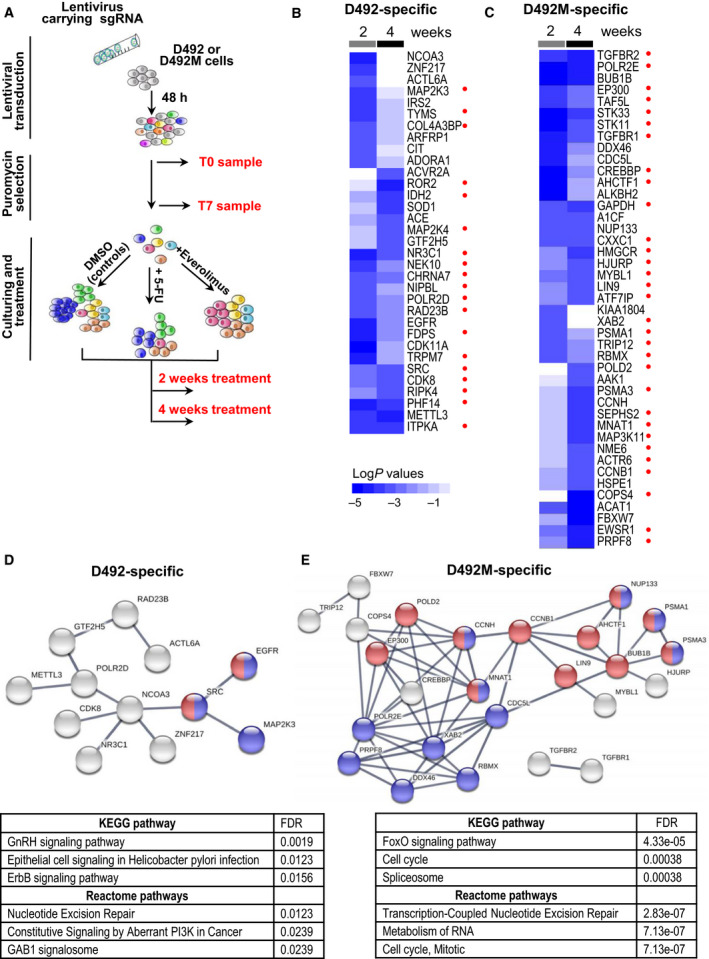
CRISPR screen‐identified gene knockouts specifically toxic to D492 or D492M cells. (A) Schematic of the CRISPR loss‐of‐function screening strategy. (B, C) Heat maps of the knockouts significantly more toxic to D492 compared with D492M (B) or D492M compared with D492 (C) with the significance level log*P* ≤ −3 in either two‐ or four‐week samples, as calculated using RSA. Color intensity indicates log*P* values. Red dots indicate selective hits that were also found using the orthogonal CRISPR analysis tool MAGeCK‐MLE. (D, E) STRING interaction network of the toxic knockouts specific for D492 (D; red and blue indicate proteins involved in ERBB and GnRH signaling, respectively) or D492M (E; red and blue indicate proteins involved in cell cycle/mitosis and RNA metabolism/spliceosome, respectively); the panels below indicate KEGG/Reactome pathways enriched in the D492‐ or D492M‐specific hit lists; FDR, false discovery rate.

We first sought to identify phenotype‐specific susceptibilities to single‐gene knockout. We employed the D492 and D492M cells and conducted a pooled CRISPR loss‐of‐function screen of 2240 genes in the ‘druggable genome’ targeting the entire human kinome, targets of FDA‐approved drugs, cancer‐related pathways, DNA damage repair genes, chromatin modifiers, and components of pathways dysregulated in tumorigenesis, tumor maintenance, and drug resistance. We employed the focused library because our hits could eventually be validated using existing targeted inhibitors, and would have a better potential for therapeutic implementation. We used lentiviral transduction to introduce barcoded sgRNA‐containing plasmids into cells, cultured for 7 days under puromycin selection to permit time for DNA editing. Then, the samples were split into treatment groups (two replicates per group) to receive DMSO vehicle or continuous drug treatment for two or four weeks. Samples were collected at time points as shown in Fig. 3A, and the number of each barcoded sgRNA remaining at each time point was counted using next‐generation sequencing (Table S1). We used the MAGeCK‐VISPR automated CRISPR screen analysis tools to determine sequencing quality (Fig. S2A–F). The Pearson correlation between sample replicates was high, and matching time points and treatments generally grouped together (Fig. S2G), revealing good reproducibility.

We first determined genetic vulnerabilities at the T7 time point, and DMSO controls at week 2 and week 4 time points. To determine the overall effectiveness and significance of each gene, taking into account the differing knockdown efficiency and activities of each of the five sgRNAs targeting it, we compared the results of two distinct analysis methods. We first used RSA to score hits based on the collective fold‐change activity of the entire sgRNA set for each gene and assign a *P*‐value for statistical significance [34]. We compared the RSA‐identified hits to those generated by MAGeCK‐MLE (Table S2). MAGeCK‐MLE automates sample normalization and comparison between T0‐, DMSO‐, and drug‐treated samples to determine a beta score, where negative beta score indicates sensitivity to loss of a gene [36]. Using either method, depletion of an sgRNA construct relative to the initial T0 condition for that cell line would indicate the loss of an essential fitness gene over time (a measure of gene ‘dependency’). If the ratio of counts for a sgRNA construct decreases between two conditions (treated *vs*. untreated or one cell line *vs*. the other), that indicates a condition‐specific essential gene.

To determine which genes were toxic in the short term when silenced, we first used RSA and identified the essential genes lost by the T7 time point for each cell line. As expected, a number of gene knockouts (such as *CDK1*, *PLK1,* and *WEE1*) centering on cell cycle, DNA repair, and RNA synthesis pathways were toxic to both cell lines (Fig. S3A). However, a large number of knockouts were selectively toxic indicating that D492 and D492M exhibit unique phenotype‐associated dependencies.

The Cancer Dependency Map (DepMap) conducted CRISPR‐Cas9 gene knockouts on hundreds of human cancer cell lines to profile their dependency on these genes (publically available at DepMap portal). We compared our most essential genes, as calculated by RSA, to the median essentiality score for each gene across all BC cell lines in DepMap (DepMap, Broad (2020): DepMap 20Q4 Public.figshare. Dataset https://doi.org/10.6084/m9.figshare.13237076.v1) [40]. We found excellent agreement for essentiality at the T7 time point (Fig. S3B,C), thus confirming our screen had identified known essential genes in BC.

We next cultured each cell line with continuous treatment of DMSO vehicle, 5‐FU, or everolimus for an additional 2 or 4 weeks to identify long‐term gene knockouts that are selectively toxic to each phenotype alone or when combined with the drug treatment. First, we compared the dependency of DMSO‐treated D492 and D492M cells as calculated by MAGeCK to the median DepMap score, and again saw a high degree of overlap as evidenced by our hits weighted toward the most essential genes in DepMap for BC (Fig. S4A). To gain further confidence that our screen was identifying known essential genes, we calculated a precision/recall plot for all DMSO‐treated conditions at both time points using the list of essential and nonessential genes published by Hart *et al*. [41] (Fig. S4B). We saw excellent precision/recall of essential and nonessential genes, as evidenced by a long straight run and linear decrease in precision as recall approaches 1.0.

We noted a strong time‐dependent effect on gene dependency. Most essential genes (those which caused a greater than twice standard deviation reduction in beta score by MAGeCK) could broadly be classified into those causing dependency at mid‐ or late time points (Fig. S5A,B and Table S3), which agrees with prior literature on the time‐dependent association with gene dependency in CRISPR screens [42]. In general, essentiality scores for D492 cells reached the highest levels, that is, lowest beta score at the mid‐time point (21 days after CRISPR knockout, the ‘Week 2’ sample) (red and green clusters in Fig. S5A). In contrast, beta scores for many essential genes in the D492M cells continued to decrease to the late time point (35 days, the ‘Week 4’ sample) (green and blue clusters in Fig. S5B). Again, this suggests differences in dependency between D492 and D492M cells. Reactome and Kegg pathway over‐representation analysis (ORA) suggested that many pathways, including cell cycle, mitotic/cell division, DNA synthesis and repair, and mRNA slicing, were more significantly enriched at the mid‐ and late time points compared with the early time point (Fig. S5C).

To discover mid/long‐term knockouts that are selectively toxic to each phenotype in the absence of drugs, we compared the gene dependencies of D492 *versus* D492M, and *vice versa*, defining hits as those with a log*P* ≤ −3 significance by RSA (Fig. 3B,C). These hits represent selective essentiality, namely that the indicated genes are specifically critical for one phenotype over the other. For increased confidence, we repeated this analysis using MAGeCK‐MLE (Fig. S6A,B), and we noted substantial overlap in the selectively essential genes identified using both methods (red marks in Fig. 3B,C and Fig. S6C–F).

Protein–protein interaction networks and KEGG/Reactome‐enriched pathways revealed vulnerability nodes specific to each phenotype (Fig. 3D,E). For D492 cells, we identified the EGFR‐SRC node, involved in GnRH signaling and ErbB signaling that were found among the top 3 KEGG pathways enriched in this dataset (Fig. 3D). Of note, EGFR was also found by RSA among the short‐term knockouts specifically toxic to D492 cells (Fig. S3A). While the MAGeCK method did not mark EGFR itself as a selective hit, the EGFR‐activated RAS‐MAPK signaling cascade was identified as a vulnerability node, with *KRAS*, *HRAS*, *RAF1*, *MAP2K1*, *MAPK1*, and *MAP2K4* */5*, all more essential to D492 cells compared with D492M (Fig. S7A). Notably, the expression level of EGFR was also considerably higher in D492 cells than D492M (Fig. S7B).

A similar analysis of the D492M‐specific hits revealed a network dominated by proteins involved in cell cycle and RNA metabolism (Fig. 3E). Genes encoding several of the highest‐scoring interacting proteins (*CCNB1*, *CCNH*, *BUB1B*, *MNAT1*) are involved in the G_2_‐M cell cycle transition. Of note, there was no significant difference in cell cycle distribution between D492 and D492M cells (Fig. S7C). Genes encoding another distinct node of interacting proteins (*PRPF8*, *DDX46*, *XAB2*, *RBMX*, *CDC5L*) are associated with RNA splicing. Among the D492M‐specific knockouts, we also found well‐known regulators of EMT, such as genes related to transforming growth factor‐beta (TGF‐β) signaling, *TGFBR1* and *TGFBR2* (Fig. 3C,E).

**Fig. 4:**
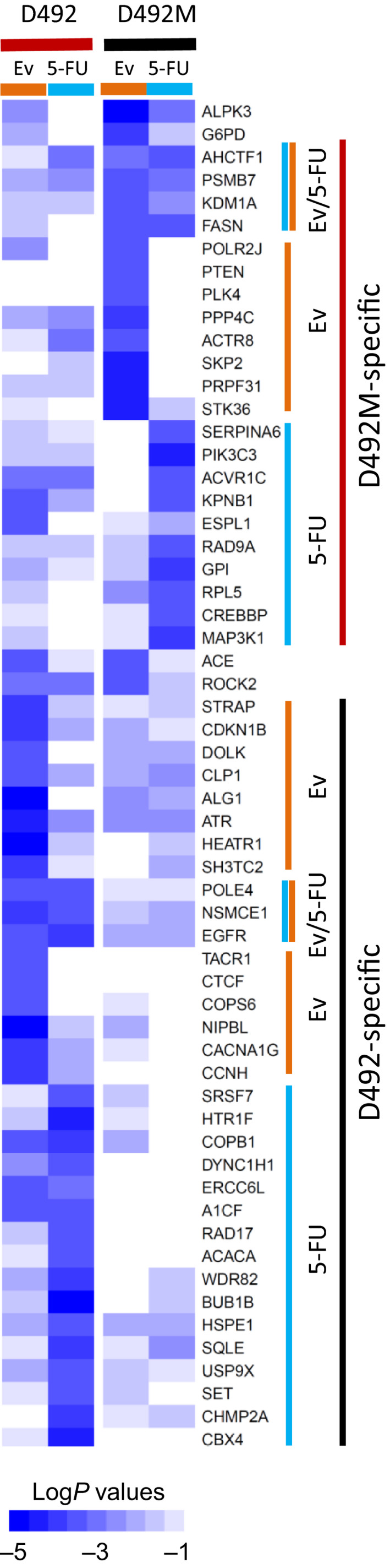
Gene knockouts sensitizing D492 or D492M cells to either 5‐FU or everolimus. Gene knockouts that are more toxic in combination with either 1 µm 5‐FU or 5 nm everolimus (Ev) after four‐week treatment; all genes where log*P* ≤ −3 by RSA in at least one condition are shown; color intensity indicates log*P* values. The gene clusters sensitizing to 5‐FU, Ev, or both (Ev/5‐FU) specific for each cell line are indicated on the right.

Next, we determined how drug treatment would affect genetic vulnerabilities. We compared 5‐FU or everolimus‐treated cells to their respective DMSO controls, revealing the knockouts that induce a greater loss of viability in the presence of the drug. We identified drug sensitizers as genes with a Log*P* ≤ −3 significance by RSA. Figure 4 shows all genes that were hits in at least one condition. In general, most hits were exclusive to either D492 or D492M cells, indicating phenotype‐dependent drug sensitization. Furthermore, in both cell lines, most of the hits were selective for 5‐FU and everolimus, suggesting a drug‐dependent sensitization. Among the few knockouts that significantly potentiated the effect of both drugs were EGFR in D492 cells and fatty acid synthase (FASN) in D492M (‘Ev/5‐FU’ clusters in Fig. 4, also found as a hit by MAGeCK, Fig. S8). Not only the knockout of EGFR itself but also several other genes related to the EGFR‐activated RAS‐MAPK signaling pathway (like *RAF1* and *MAPK1*) potentiated the drugs more significantly in D492 cells compared with D492M (Fig. S9). Regarding FASN, our RPPA analysis revealed threefold higher expression of FASN protein in D492M cells compared with D492 (Fig. S10). FASN was also found among the D492M‐specific short‐term essential genes (Fig. S3A). Collectively, this suggests the importance of FASN for D492M cells, particularly under ‘therapy pressure’.

### 3.4 Phenotype‐specific genetic vulnerabilities represent actionable targets

To confirm that the identified genetic vulnerabilities represent phenotype‐specific targets for therapy, we tested the effect of inhibitors of EGFR, cell cycle progression, and FASN alone and in combination with everolimus or 5‐FU. We used 3D cell cultures in Matrigel that are considered to be a more representative model for testing therapies and where prolonged, 8‐day treatment could be performed [43].

**Fig. 5:**
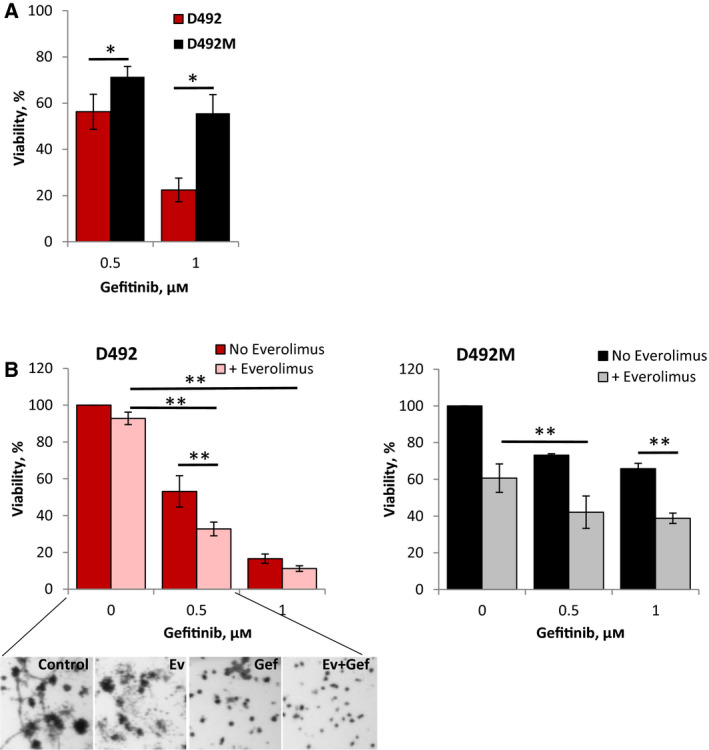
Sensitivity of D492 and D492M cells to the EGFR inhibitor gefitinib. The cells were grown in 3D Matrigel and treated for 8 days with the indicated concentrations of gefitinib alone (A) or in combination with 5 nm everolimus (B) before the cell survival was measured by the CTG method; average ± SEM [*n* ≥ 6 (A), *n* = 3 (B)]; the representative pictures of the D492 cell colonies are shown in the lower panel; * and **, *P* ≤ 0.05 by unpaired and paired *t*‐test, respectively.

To target EGFR, we applied gefitinib, a clinically approved inhibitor of the EGFR tyrosine kinase domain. Gefitinib alone induced a dose‐dependent response and was significantly more potent in D492 compared with D492M cells (Fig. 5A and Fig. S11A). Of note, D492 cells were also more sensitive to the ERK inhibitor SCH772984 that targets the MAPK signaling pathway downstream from EGFR (Fig. S11B). Inhibition of EGFR in the presence of everolimus resulted in a significantly higher antiproliferative effect than either treatment alone, and the total effect was stronger in D492 cells than in D492M (Fig. 5B). Similar results were observed when gefitinib was combined with 5‐FU (Fig. S12A). Thus, pharmacological inhibition of EGFR or ERK impacted cell viability in line with the CRISPR results, suggesting that EGFR‐RAS‐MAPK signaling cascade is more important for the epithelial phenotype cells in the absence and presence of ‘therapy pressure’.

**Fig. 6:**
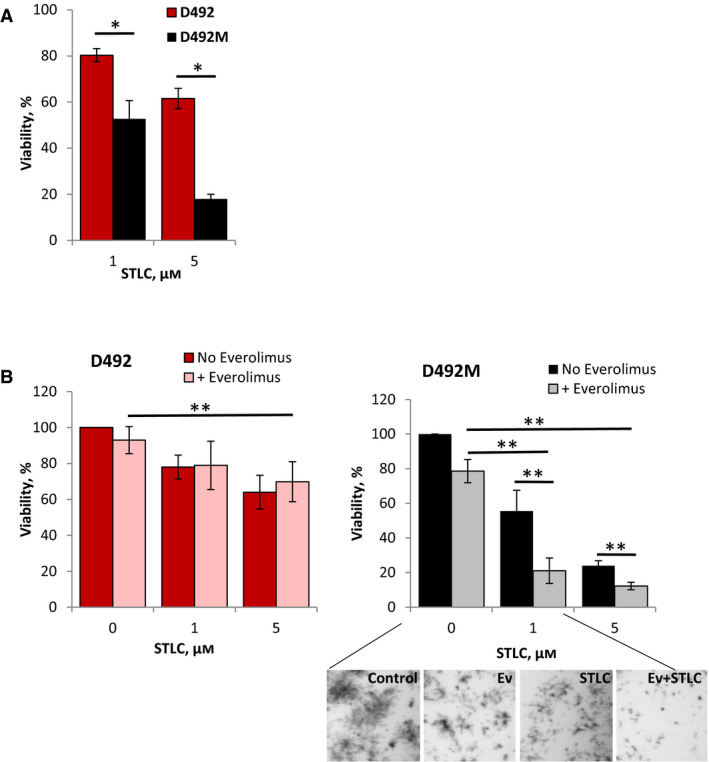
Sensitivity of D492 and D492M cells to the G_2_‐M inhibitor STLC. The cells were grown in 3D Matrigel and treated for 8 days with the indicated concentrations of STLC alone (A) or in combination with 5 nm everolimus (B) before the cell survival was measured by the CTG method; average ± SEM [*n* ≥ 6 (A), *n* ≥ 3 (B)]; the representative pictures of the D492M cell colonies are shown in the lower panel; * and **, *P* ≤ 0.05 by unpaired and paired *t*‐test, respectively.

To block the G_2_‐M cell cycle transition, we used an inhibitor of kinesin 5 (EG5). Although EG5 was not included in the CRISPR screen, EG5 is a critical mediator of the G_2_‐M transition and therefore a good target to disrupt this process. EG5 drives the microtubule assembly during the mitotic spindle formation [44], and EG5 inhibitors, such as *S*‐trityl‐l‐cysteine (STLC), prevent correct spindle organization, leading to the cell cycle arrest in G_2_‐M [45]. Here, we show that D492M cells are more sensitive to the treatment with STLC than D492 cells (Fig. 6A). Furthermore, in D492M cells a combination of STLC and everolimus resulted in a greater antiproliferative effect compared with either treatment alone, which was not the case in D492 cells (Fig. 6B). However, we did not observe a clear benefit of adding STLC to 5‐FU (Fig. S12B). This could be explained by the fact that 5‐FU acts on dividing cells, and STLC interrupts cell division. Taken together, these findings suggest that the EG5 inhibition selectively impacts growth in the mesenchymal‐like cells and may sensitize them to targeted drugs, such as everolimus.

**Fig. 7:**
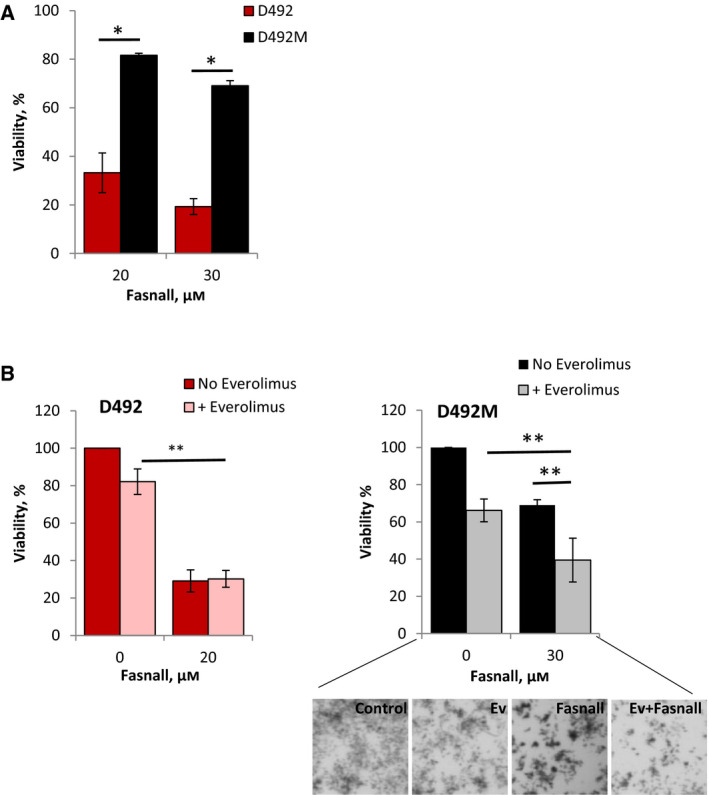
Sensitivity of D492 and D492M cells to the FASN inhibitor Fasnall. The cells were grown in 3D Matrigel and treated for 8 days with Fasnall alone (A) or in combination with 5 nm everolimus (B) before the cell survival was measured by the CTG method; average ± SEM (*n* ≥ 3) except for 20 µm in A, where ± SD (*n* = 2); the representative pictures of the D492M cell colonies are shown in the lower panel; * and **, *P* ≤ 0.05 by unpaired and paired *t*‐test, respectively.

To target FASN, we used Fasnall, a selective inhibitor that has shown a potent antitumor activity in BC models [46]. FASN knockout was identified as a sensitizer to the drugs in D492M cells, and FASN protein expression was higher in D492M, suggesting the importance of FASN for survival of the mesenchymal cells. However, we did not observe a higher sensitivity to FASN inhibition with Fasnall in D492M cells. On the contrary, D492 cells were more vulnerable to the used doses of Fasnall (Fig. 7A). However, in line with the CRISPR screen results, the antiproliferative effect of Fasnall in D492M was enhanced by combination with the drugs (Fig. 7B and Fig. S12C), whereas there was no significant improvement in D492 cells.

In summary, by applying a CRISPR‐Cas9 loss‐of‐function screen, we detected phenotype‐specific vulnerability genes in the absence or presence of clinically relevant therapy. The identified genetic vulnerabilities pinpoint actionable targets that can be used to design combinatorial treatments to target cells of distinct phenotypes.

## 4 Discussion

The goal of this study was to detect therapeutic vulnerabilities unique to either the epithelial or mesenchymal phenotype in breast epithelial cells by using a CRISPR‐Cas9 genetic loss‐of‐function screen. Previous studies have employed a similar approach in a range of cancer cell lines to identify genotype‐specific vulnerabilities and promising therapeutic targets [40, 47, 48, 49]. To the best of our knowledge, this is the first study that employs such a screen in isogenic cell lines displaying opposing phenotypes on the EMT spectrum.

Both the CRISPR screen and the validation analyses with targeted inhibitors suggest that the epithelial phenotype is more sensitive to disruption of EGFR and the downstream RAS‐MAPK signaling pathway, while the mesenchymal phenotype is more susceptible to disruption of the G_2_‐M cell cycle transition. The association between EGFR and the epithelial phenotype has been observed previously, where introduction of EGFR into the mesenchymal‐like D492 cells reversed them into the epithelial state [50]. Furthermore, computational approaches predicted a higher flux through the EGFR and RAS‐MAPK signaling network in D492 cells, and the genes from these pathways were predicted to reverse D492M into D492 phenotype [51]. It has also been demonstrated that acquisition of the mesenchymal features reduces sensitivity to EGFR inhibitors [52, 53]. This is in line with our observation that gefitinib was more potent in D492 cells than D492M. Furthermore, EGFR knockout and gefitinib sensitized D492 cells to everolimus or 5‐FU leading to a stronger antiproliferative effect than what was achieved in D492M. Taken together, these results suggest that the epithelial phenotype is dependent on EGFR and the downstream RAS‐MAPK signaling pathway that represent therapeutic vulnerabilities and could be exploited as targets in combination therapies.

Identification of the G_2_‐M transition as a vital node in the mesenchymal phenotype also agrees with the previous studies. It has been reported that G_2_‐M arrest by the anti‐microtubule drug paclitaxel or inhibition of aurora kinases prevented mesenchymal transition [54, 55]. Furthermore, we also identified loss of *AURKA*, encoding the aurora kinase A, to be more toxic to the D492M cells than D492 (Table S3). Based on these data, further investigation of clinically relevant G_2_‐M inhibitors for targeting the mesenchymal phenotype could be suggested. Of note, a clinical trial, where the aurora kinase A‐selective inhibitor alisertib was used in combination with paclitaxel, revealed a therapeutic benefit of adding alisertib in advanced BC [56].

In addition, we identified knockouts of TGF‐β receptors (*TGFBR*s) and RNA splicing machinery as selectively toxic for the mesenchymal‐like cells. These observations are in line with what is known about their association with EMT. TGF‐β is a known inducer of EMT in advanced cancers [57]. Furthermore, increase in TGFBR expression and signaling has been linked to taxane resistance, and treatment with TGFBR inhibitors could prevent both EMT and chemoresistance in breast cancer [58]. Notably, EMT has also been linked to alternative splicing in breast cancer [59]. Although it is an interesting observation with potential prognostic value, it does not represent a therapeutically tractable target.

In search of genes whose loss is selectively toxic to the mesenchymal phenotype under ‘therapy pressure’, we identified FASN. By combining the FASN inhibitor Fasnall with everolimus, we confirmed this finding in D492M cells. FASN is an attractive therapeutic target, since it is overexpressed in tumor cells compared with normal cells [60]. FASN modulates cellular energetics and membrane architecture, thereby influencing oncogenic signaling and sustaining cancer cell growth and survival [61]. FASN expression has been shown to correlate with BC progression, resistance, and poor prognosis [62]. In this respect, high FASN levels are expected to be linked to EMT/mesenchymal phenotype, as shown by others [63], and also detected in the D492M cells. The latter coincides with the higher flux of fatty acid oxidation and the different lipid profile in D492M cells, as shown previously [64, 65]. Altogether, this suggests that EMT is associated with changes in lipid metabolism and that cells of the mesenchymal phenotype are sensitive to intervention within this metabolic system. In contrast to what could be expected, we detected lower sensitivity to the FASN inhibitor Fasnall in D492M cells than D492. This might be due to higher endogenous expression of the FASN protein and, thus, incomplete pharmacological inhibition in D492M cells. Notably, FASN knockout was also found among the D492M‐specific short‐term vulnerabilities, arguing for FASN as an actionable target in the mesenchymal phenotype, particularly in combination with clinically used anticancer drugs.

The findings presented here are based on D492 and D492M cells that are nontumorigenic. However, D492 cell model shows similarities to TNBC, due to their native basal cell phenotype, stem cell‐like properties, lack of expression of hormone receptors, and HER2 and ability to undergo EMT. Therefore, our findings could be relevant to TNBC that demonstrates high degree of phenotypic heterogeneity and could benefit from phenotype‐tailored combination therapies.

## 5 Conclusion

Our findings support the application of a CRISPR genetic loss‐of‐function screen as an effective strategy to identify phenotype‐specific therapeutic vulnerabilities. The disclosed genetic vulnerabilities pinpoint actionable targets and suggest inhibitors for combination therapies that can target distinct phenotypes.

## Author contributions

AB, CMG, BH, CJD, SAM, OE, GMM, and LP designed the study. AB, CMG, KS, SP, CS, and EB performed experiments and data analysis. TG provided the cells and protocols for their culturing and analyses. AB, CMG, and LP wrote the manuscript. All authors critically evaluated the results and the manuscript.

## Conflict of interest

CJD is on the Scientific Advisory Board of Anchiano Therapeutics, Mirati Therapeutics, and Oncogenuity, and is a consultant for Deciphera Pharmaceuticals, Eli Lilly, Jazz Therapeutics, Robometrix, Sanofi, SpringWorks Therapeutics, and Turning Point Therapeutics. CJD has received support from Mirati Therapeutics, Deciphera Pharmaceuticals, and SpringWorks Therapeutics. All other authors have no competing financial and/or nonfinancial interests in relation to this work.

## Supporting information


**Fig. S1.** Effect of 5‐FU on cell growth and the level of mTOR signaling‐related proteins. A, Cell confluence (tracked by Incucyte, left panel) in D492 cultures in 2D treated with indicated concentration of 5‐FU; right panel: relative cell density in the treated samples normalized to the nontreated controls (set to 100); average +/‐SEM (n = 6); * p ≤ 0.05, unpaired t‐test; B, Relative levels of mTOR and its downstream target (phospho)proteins as detected by RPPA in D492 and D492M cells; average +/‐ StDev from three technical parallels.

**Fig. S2.** CRISPR screen sequencing and replicate quality analysis. A, Distribution of mean sequence quality. B, Distribution of GC content. C, Mapping ratio of all reads for indicated conditions. D, Gini index. E, Number of zero‐count sgRNAs per sample. F, normalized read count distribution, plotted as mean with 10‐90 percentile whiskers. G, Heat map of Pearson correlation scores for all CRISPR screen samples with unsupervised clustering.

**Fig. S3.** Short‐term vulnerability genes in D492 and D492M cells overlap with DepMap dependency. A, Short‐term gene essentiality was determined using RSA by taking the ratio of sgRNA construct representation at the T7 time point compared to T0. All genes where the logP significance was ≤‐2 are shown (EGFR and FASN are indicated); color intensity indicates logP values. B and C, The median DepMap gene essentiality score with CRISPR knockout (CERES score) across breast cancer cell lines is shown for every gene in the CRISPR library. Negative scores indicate increasing dependency on that gene. Red highlighted points are essential genes identified in (A) for the indicated cell line.

**Fig. S4.** Mid/long‐term vulnerability genes in D492 and D492M cells compared to the Cancer Dependency Map. A, DepMap CERES gene dependency scores shown for each gene in the CRISPR library. Red highlighted points indicate genes found to be essential to the indicated cell lines at either week two or week four, with hits defined as genes having scores greater than three standard deviations from the mean using MAGeCK‐MLE. B, Precision/recall plot for D492 and D492M cells after two or four weeks DMSO treatment of previously published in Hart et al. [41] known essential genes compared to non‐essential genes.

**Fig. S5.** Time influence on gene essentiality. A and B, Genes in D492 (A) and D492M (B) cells were defined as ‘essential’ if their beta score decreased by two or more standard deviations from the mean at an early (7d), mid (21d), or late (35d) time point. Using unsupervised clustering, genes separated into distinct groups: mid‐(highly)essential, where gene dependency reached maximum at day 21 and did not change further with time; late‐(highly)essential, where dependency increased with time and reached maximum at the final time point, day 35d; the ‘variable/weak essential’ cluster included genes, whose essentiality was observed only at a single time point, was weak and did not increase over time. C, Representative enriched Reactome and Kegg pathways using Over‐Representation Analysis (ORA) for D492 and D492M cell lines at indicated time points.

**Fig. S6.** Phenotype‐selective genes identified from CRISPR screen using MAGeCK‐MLE. A and B, After two weeks (A) or four weeks (B) of DMSO vehicle treatment, sgRNAs targeting indicated genes may be either depleted or enriched. Phenotype‐selective gene essentiality consists of genes with greater depletion in one cell line compared to the other. Shown is the beta score as calculated using MAGeCK‐MLE. The top 15 genes in each category are labeled. C‐F, Beta scores given by MAGeCK‐MLE for indicated conditions shown with RSA hits (logP ≤ −3) highlighted in red, which are weighted towards the more essential genes as ranked by MAGeCK‐MLE. Magenta‐highlighted genes are previously published non‐essential genes [41].

**Fig. S7.** Comparison of the significance of the EGFR signaling‐related gene knockouts, EGFR protein levels and cell cycle distribution in D492 and D492M cells. A, Heat map of MAGeCK‐MLE results for D492 and D492M cells for EGFR‐RAS‐MAPK signaling related genes. B, Immunofluorescence pictures indicating EGFR protein level; scale bar, 20 µm. C, DNA content indicating cell cycle distribution in D492 and D492M cells as detected by flow cytometry.

**Fig. S8.** Phenotype‐specific gene cooperativity with everolimus and 5‐FU identified using MAGeCK‐MLE. CRISPR hits for everolimus (A, C) and 5‐FU (B, D) treated cells in indicated cell lines. Phenotype‐specific gene essentiality consists of genes with greater depletion in one cell line compared to the other. Shown is the beta score as calculated using MAGeCK‐MLE. The top 15 genes in each category are labeled.

**Fig. S9.** The significance of EGFR signaling‐related gene knockouts for toxicity in cells under ‘therapy pressure’. LogP values for EGFR signaling related genes (defined by Ingenuity Pathway Analysis) in everolimus‐ or 5‐FU‐ treated *versus* nontreated D492 or D492M cells at week two or week four.

**Fig. S10.** FASN protein expression in D492 and D492M cells. FASN expression level was detected by RPPA (average +/‐ StDev from three technical parallels).

**Fig. S11.** Sensitivity of 2D cultures of D492 and D492M to the EGFR and ERK inhibitors. Cells were grown as monolayers in 2D and treated for three days with the indicated concentrations of the EGFR inhibitor gefitinib (A) or the ERK inhibitor SCH772984 (B) before the cell survival was measured by the CTG method; average +/‐ SEM (n ≥ 4); *, p ≤ 0.05 by unpaired t‐test.

**Fig. S12.** Sensitivity of D492 and D492M cells to gefitinib, STLC and Fasnall with/without additional treatment with 5‐FU. The cells were grown in 3D Matrigel and treated for eight days with 1 µM gefitinib (A), 5 µM (D492) or 1 µM (D492M) STLC (B) or 20 µM (D492) or 30 µM (D492M) Fasnall (C) in combination with 1 µM 5‐FU before the cell survival was measured by the CTG method; average +/‐ SEM (n = 3); ** p ≤ 0.05 by paired t‐test.

**Table S1.** Sequencing counts for each sgRNA construct for each condition.

**Table S2.** MAGeCK‐MLE gene summary analysis results.

**Table S3.** Essential genes in the D492 and D492M cells at indicated days post‐infection. Beta scores for each gene are provided at each time point in a cell line if that gene was essential in that cell line at any time point. Genes were defined as ‘essential’ if their beta score decreased by two or more standard deviations from the mean at an early (7 d), mid (21 d) or late (35 d) time point.

## Data Availability

The data that support the findings of this study are available in the Supporting Information (Tables [Link], [Link], [Link]) of this article.
