# Supplementary figures and images for: Detection of phenotype‐specific therapeutic vulnerabilities in breast cells using a CRISPR loss‐of‐function screen
Source: Mol Oncol. 2021 May 1;15(8):2026–45. doi: 10.1002/1878-0261.12951 (PMC8333781; doi:10.1002/1878-0261.12951)

# Supplementary Figure S1

**A**

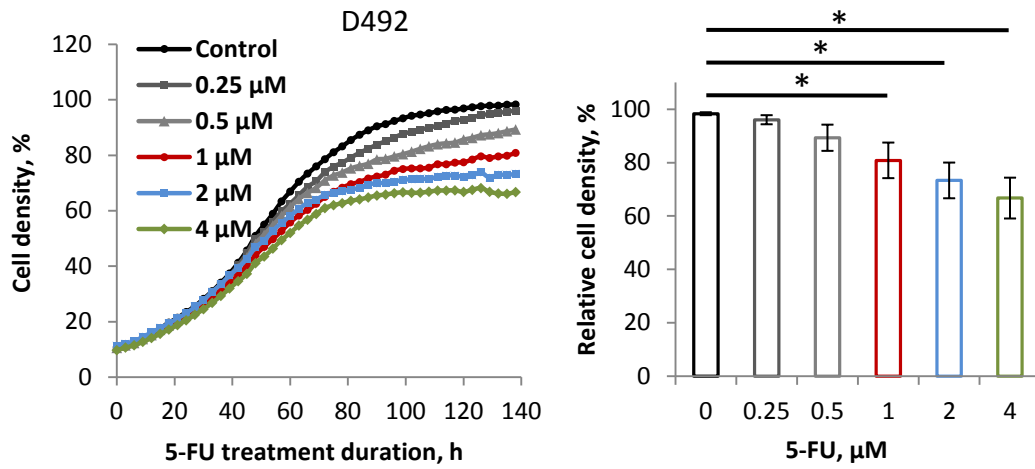

**B**

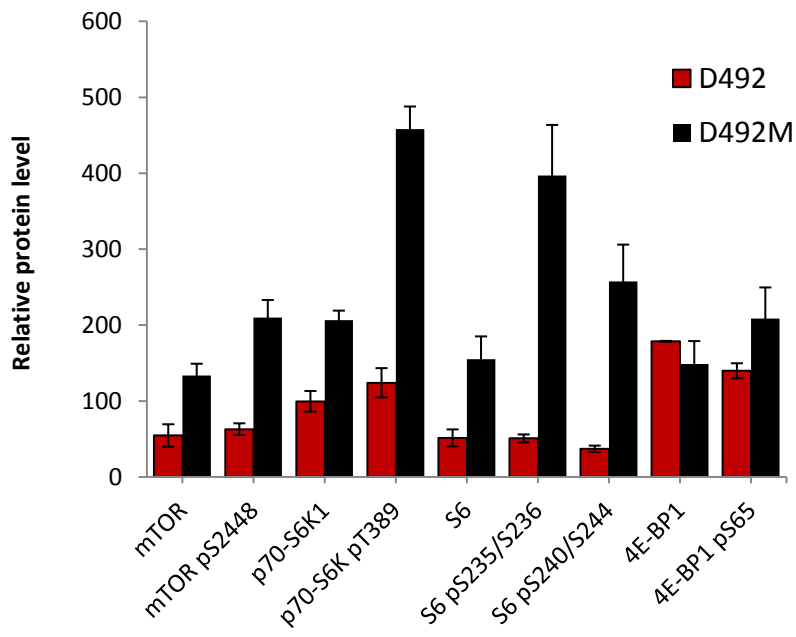

Supplement: Supplementary file 1 — Fig. S1. Effect of 5‐FU on cell growth and the level of mTOR signaling‐related proteins. A, Cell confluence (tracked by Incucyte, left panel) in D492 cultures in 2D treated with indicated concentration of 5‐FU; right panel: relative cell density in the treated samples normalized to the nontreated controls (set to 100); average +/‐SEM (n = 6); * p ≤ 0.05, unpaired t‐test; B, Relative levels of mTOR and its downstream target (phospho)proteins as detected by RPPA in D492 and D492M cells; average +/‐ StDev from three technical parallels. [file MOL2-15-2026-s004.pdf]

A

D492 D492M

## Supplementary Figure S3

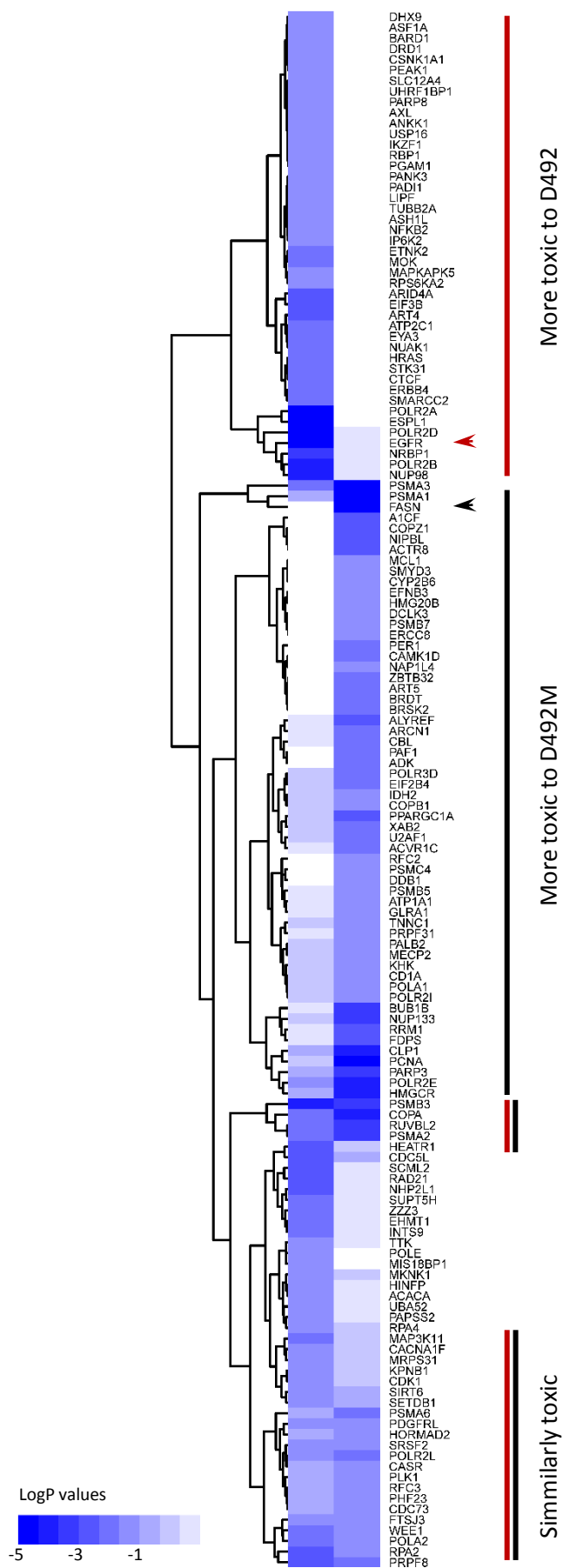

**B**

**D492 T7**

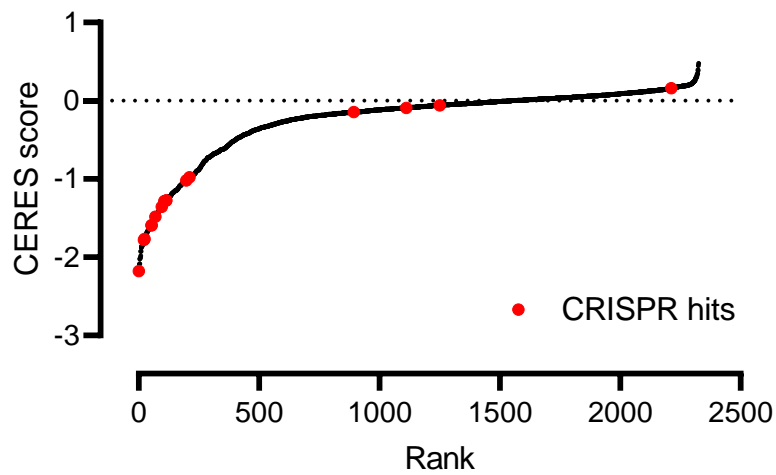

**C**

**D492M T7**

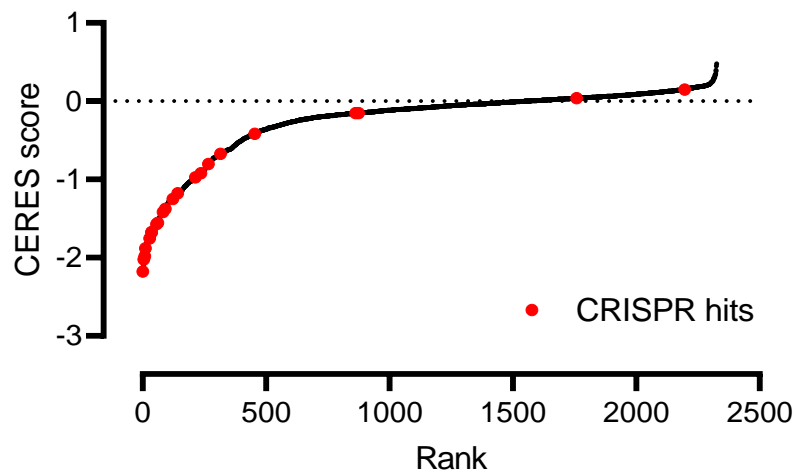

Supplement: Supplementary file 3 — Fig. S3. Short‐term vulnerability genes in D492 and D492M cells overlap with DepMap dependency. A, Short‐term gene essentiality was determined using RSA by taking the ratio of sgRNA construct representation at the T7 time point compared to T0. All genes where the logP significance was ≤‐2 are shown (EGFR and FASN are indicated); color intensity indicates logP values. B and C, The median DepMap gene essentiality score with CRISPR knockout (CERES score) across breast cancer cell lines is shown for every gene in the CRISPR library. Negative scores indicate increasing dependency on that gene. Red highlighted points are essential genes identified in (A) for the indicated cell line. [file MOL2-15-2026-s008.pdf]

# Supplementary Figure S4

**A**

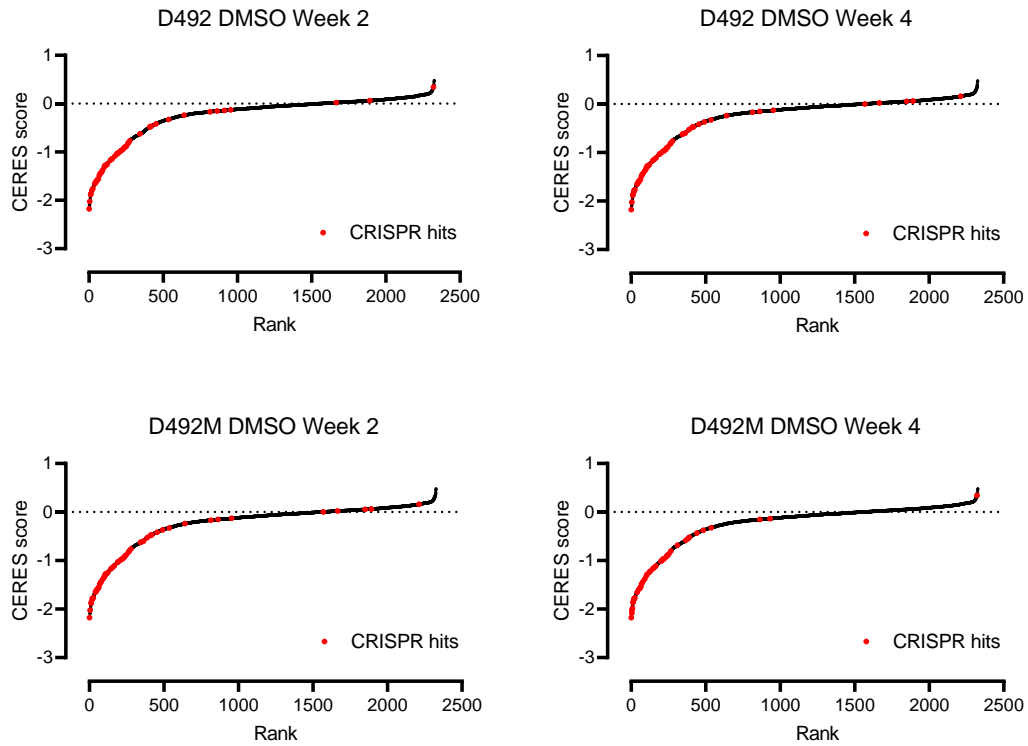

**B**

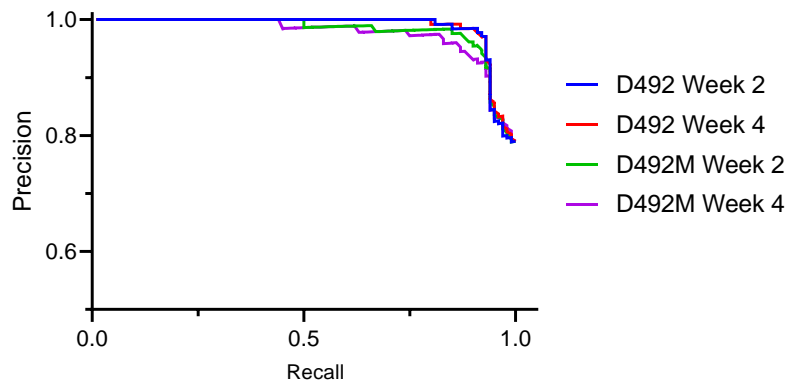

Supplement: Supplementary file 4 — Fig. S4. Mid/long‐term vulnerability genes in D492 and D492M cells compared to the Cancer Dependency Map. A, DepMap CERES gene dependency scores shown for each gene in the CRISPR library. Red highlighted points indicate genes found to be essential to the indicated cell lines at either week two or week four, with hits defined as genes having scores greater than three standard deviations from the mean using MAGeCK‐MLE. B, Precision/recall plot for D492 and D492M cells after two or four weeks DMSO treatment of previously published in Hart et al. [41] known essential genes compared to non‐essential genes. [file MOL2-15-2026-s005.pdf]

**A**

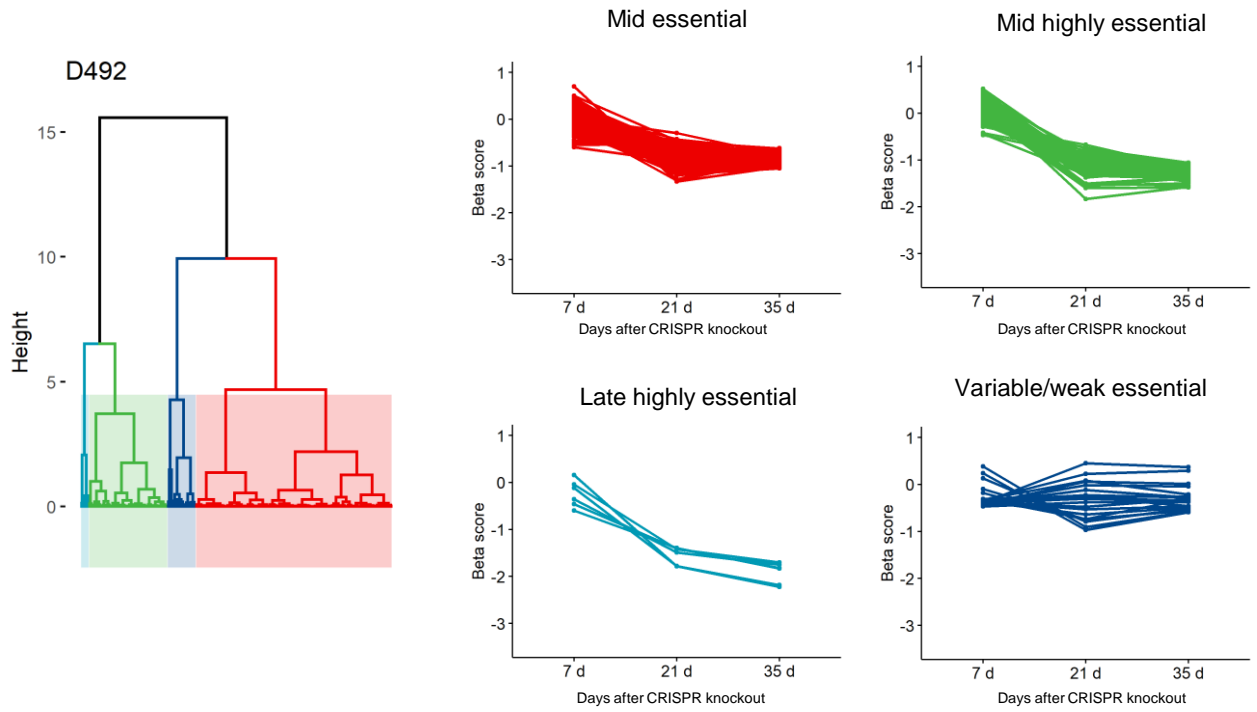

**B**

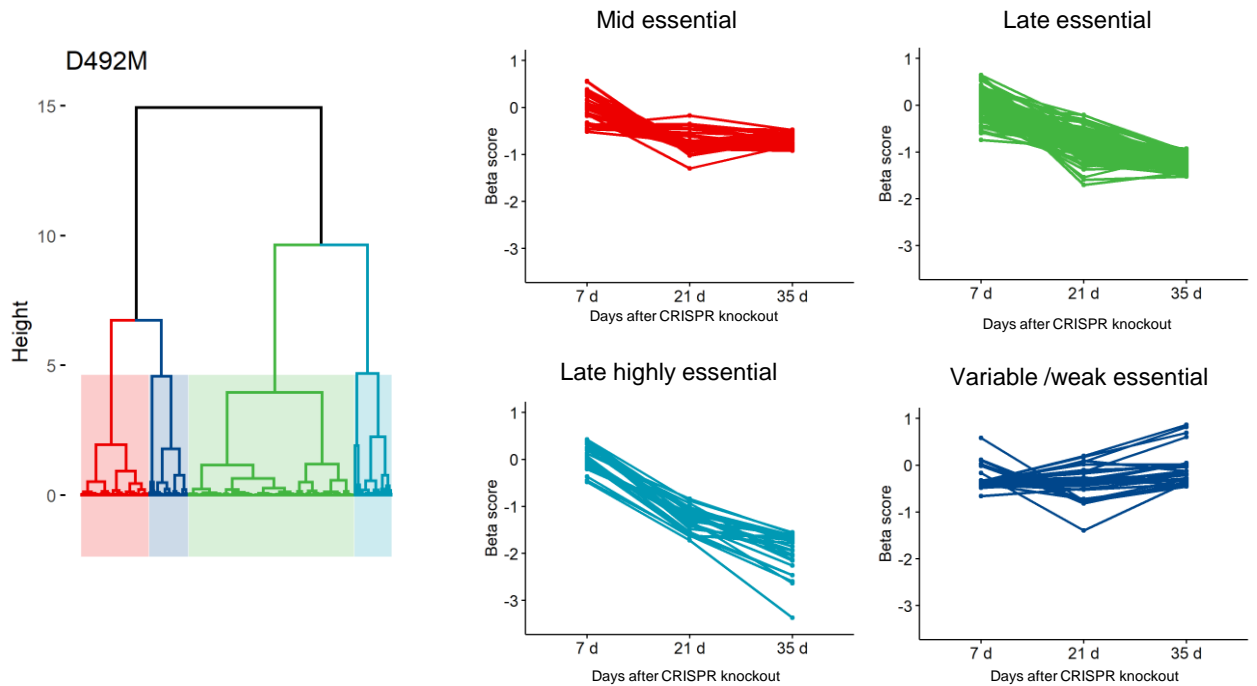

# Supplementary Figure S5 (cont)

C

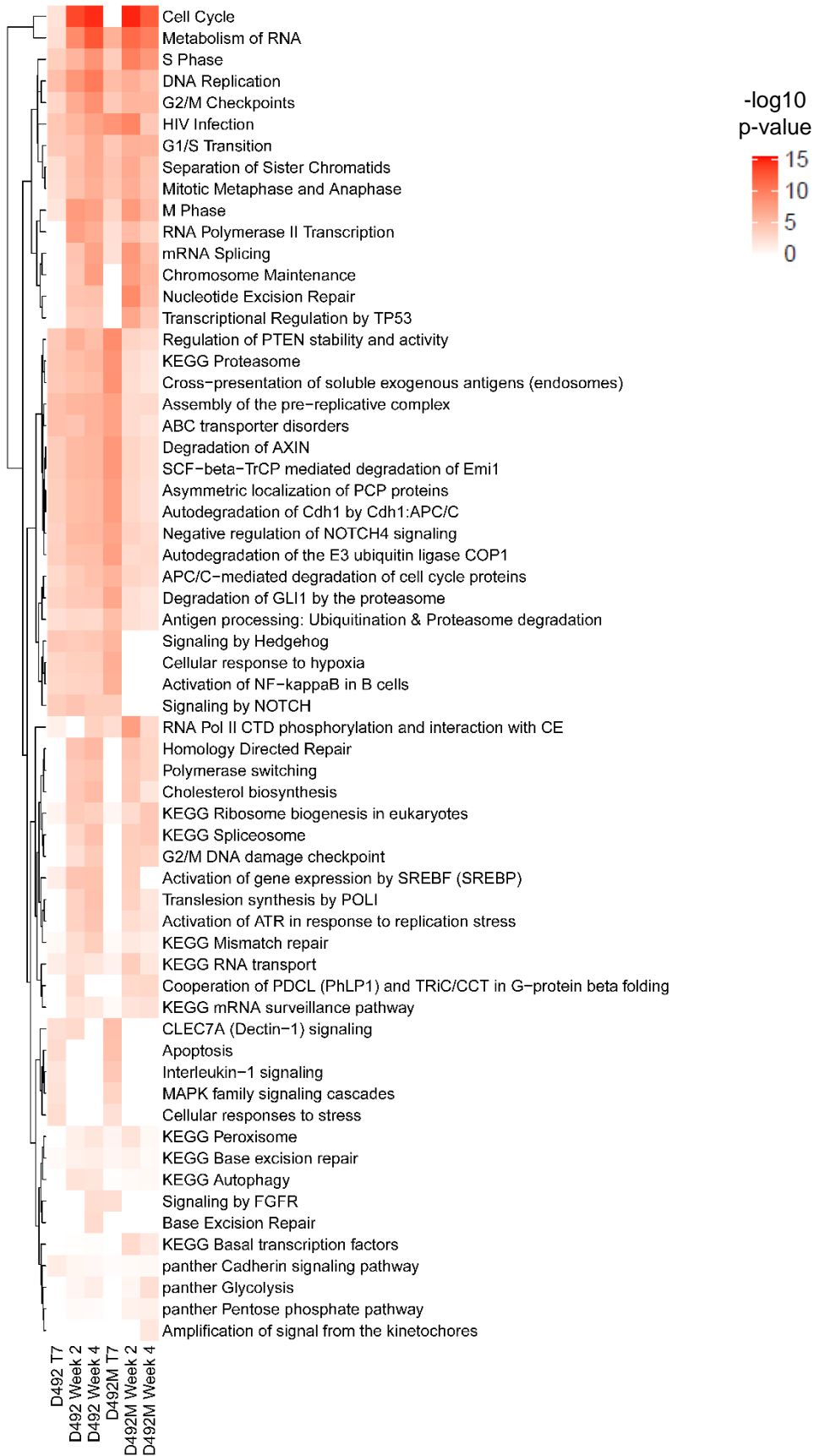

Supplement: Supplementary file 5 — Fig. S5. Time influence on gene essentiality. A and B, Genes in D492 (A) and D492M (B) cells were defined as ‘essential’ if their beta score decreased by two or more standard deviations from the mean at an early (7d), mid (21d), or late (35d) time point. Using unsupervised clustering, genes separated into distinct groups: mid‐(highly)essential, where gene dependency reached maximum at day 21 and did not change further with time; late‐(highly)essential, where dependency increased with time and reached maximum at the final time point, day 35d; the ‘variable/weak essential’ cluster included genes, whose essentiality was observed only at a single time point, was weak and did not increase over time. C, Representative enriched Reactome and Kegg pathways using Over‐Representation Analysis (ORA) for D492 and D492M cell lines at indicated time points. [file MOL2-15-2026-s012.pdf]

### Supplementary Figure S6

**A**

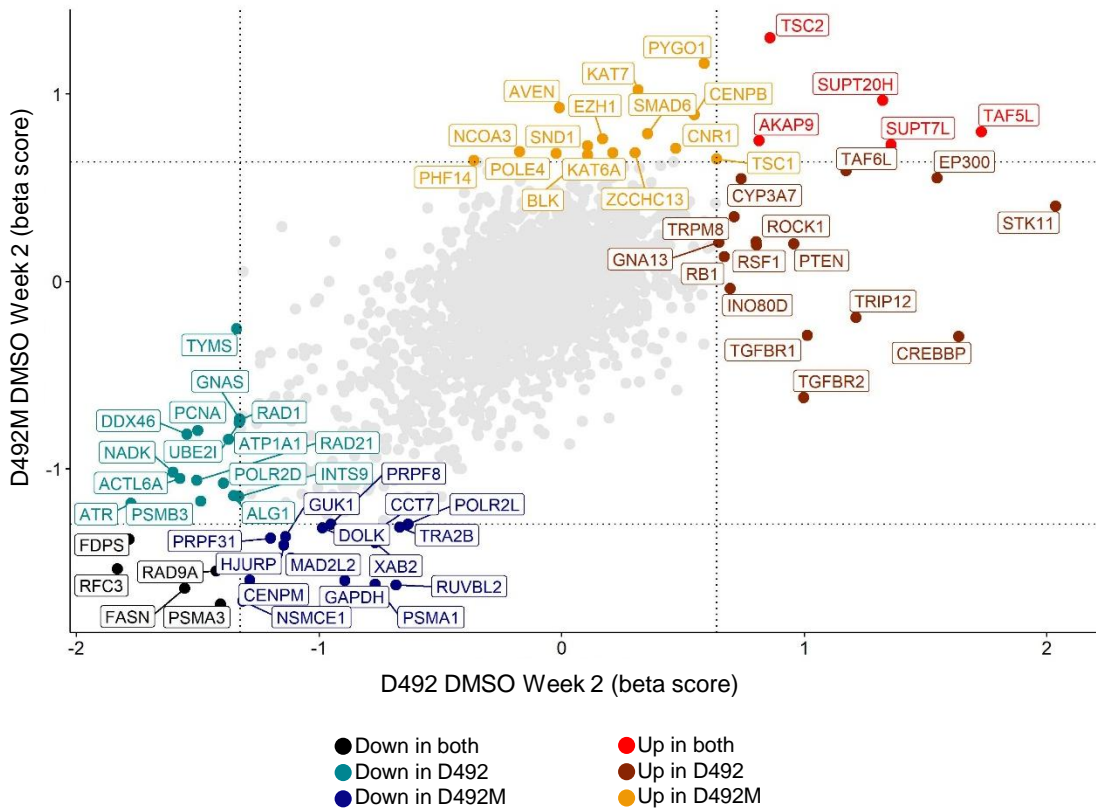

**B**

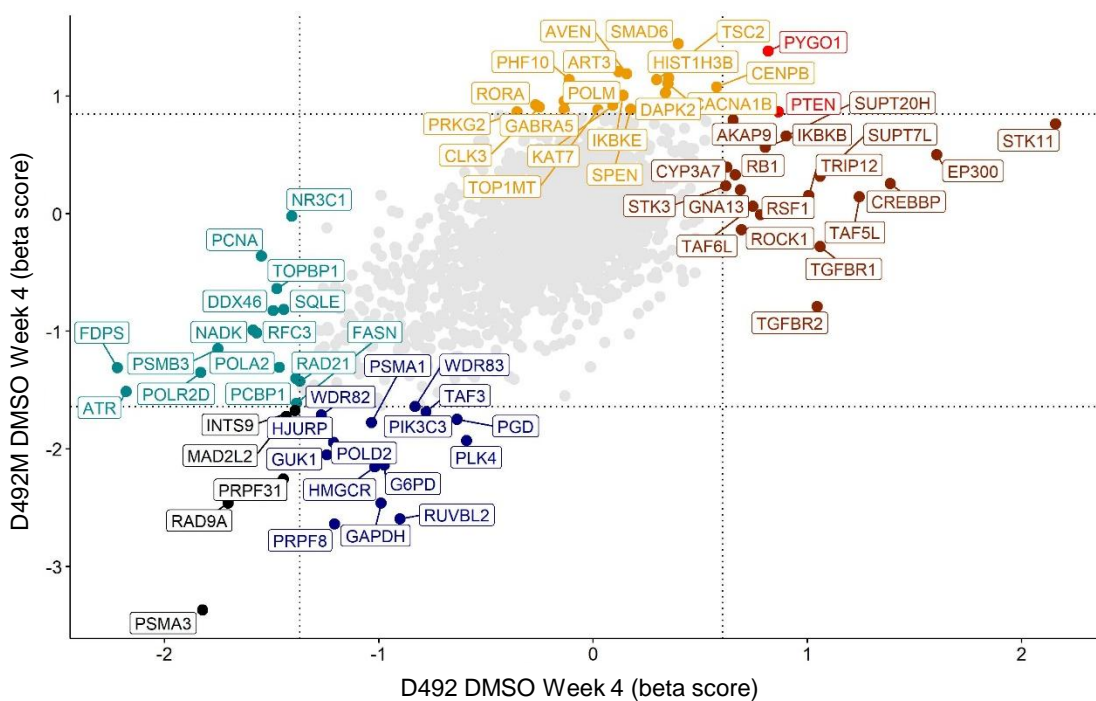

## Supplementary Figure S6 (cont)

**C**

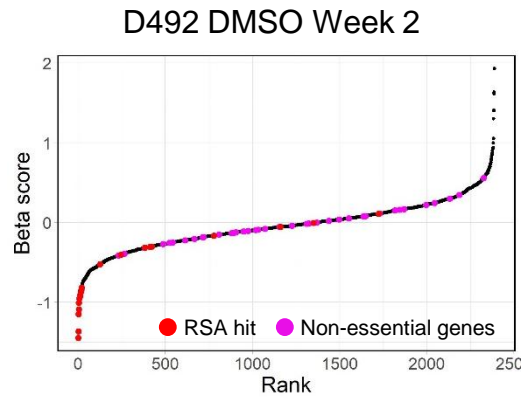

**D**

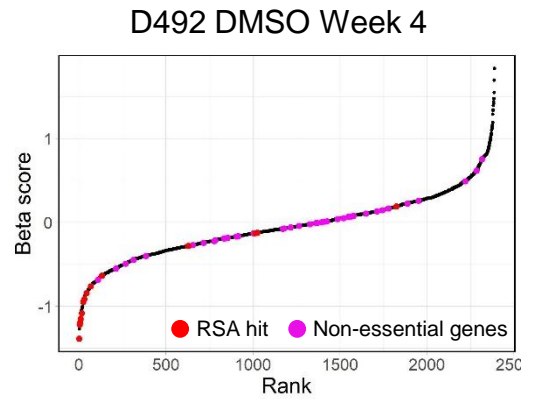

**E**

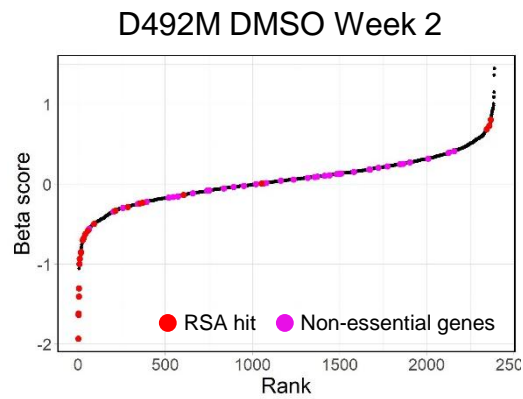

**F**

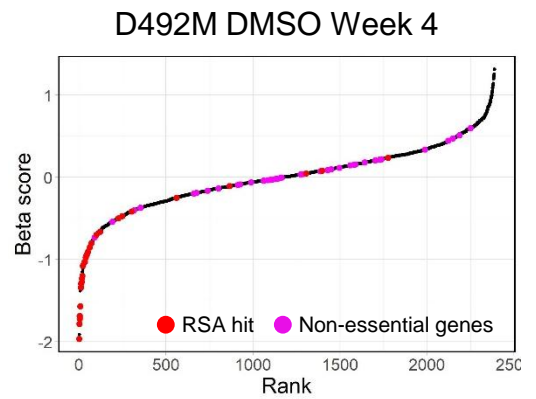

Supplement: Supplementary file 6 — Fig. S6. Phenotype‐selective genes identified from CRISPR screen using MAGeCK‐MLE. A and B, After two weeks (A) or four weeks (B) of DMSO vehicle treatment, sgRNAs targeting indicated genes may be either depleted or enriched. Phenotype‐selective gene essentiality consists of genes with greater depletion in one cell line compared to the other. Shown is the beta score as calculated using MAGeCK‐MLE. The top 15 genes in each category are labeled. C‐F, Beta scores given by MAGeCK‐MLE for indicated conditions shown with RSA hits (logP ≤ −3) highlighted in red, which are weighted towards the more essential genes as ranked by MAGeCK‐MLE. Magenta‐highlighted genes are previously published non‐essential genes [41]. [file MOL2-15-2026-s011.pdf]

# Supplementary Figure S7

**A**

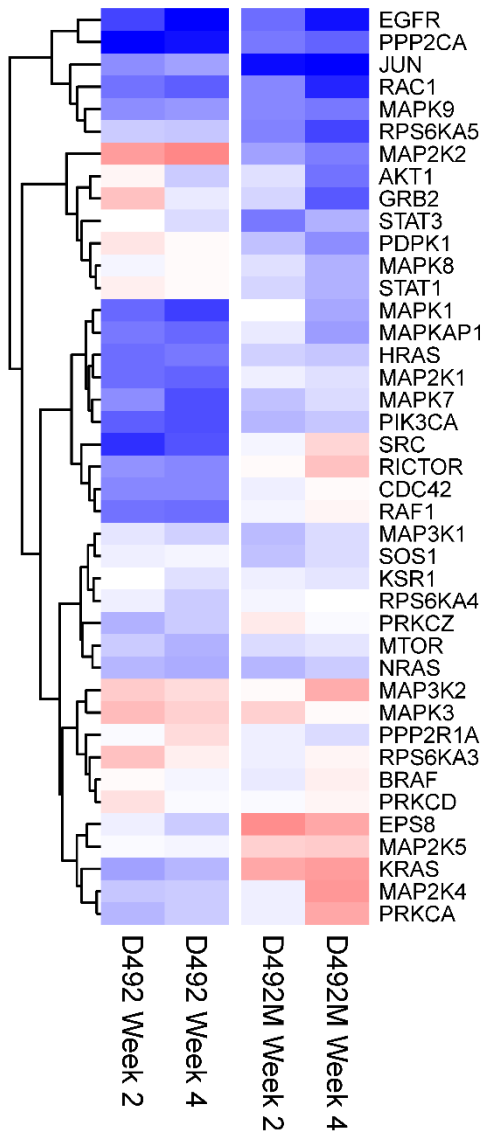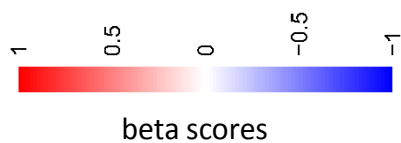

**B**

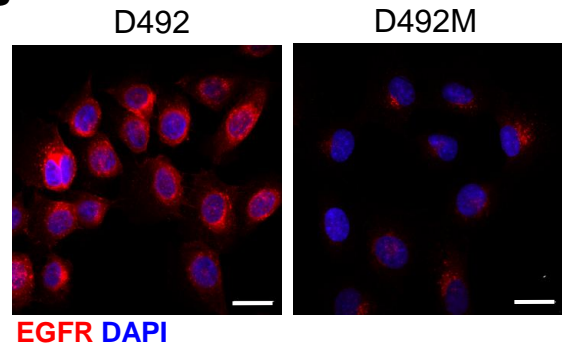

**C**

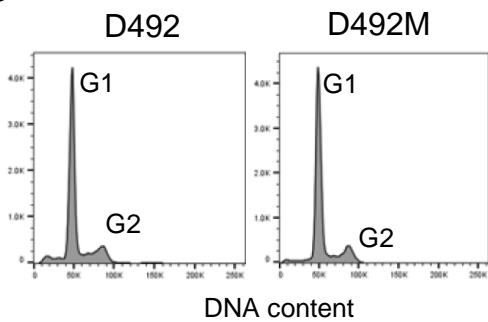

Supplement: Supplementary file 7 — Fig. S7. Comparison of the significance of the EGFR signaling‐related gene knockouts, EGFR protein levels and cell cycle distribution in D492 and D492M cells. A, Heat map of MAGeCK‐MLE results for D492 and D492M cells for EGFR‐RAS‐MAPK signaling related genes. B, Immunofluorescence pictures indicating EGFR protein level; scale bar, 20 µm. C, DNA content indicating cell cycle distribution in D492 and D492M cells as detected by flow cytometry. [file MOL2-15-2026-s003.pdf]

### Supplementary Figure S8

**A**

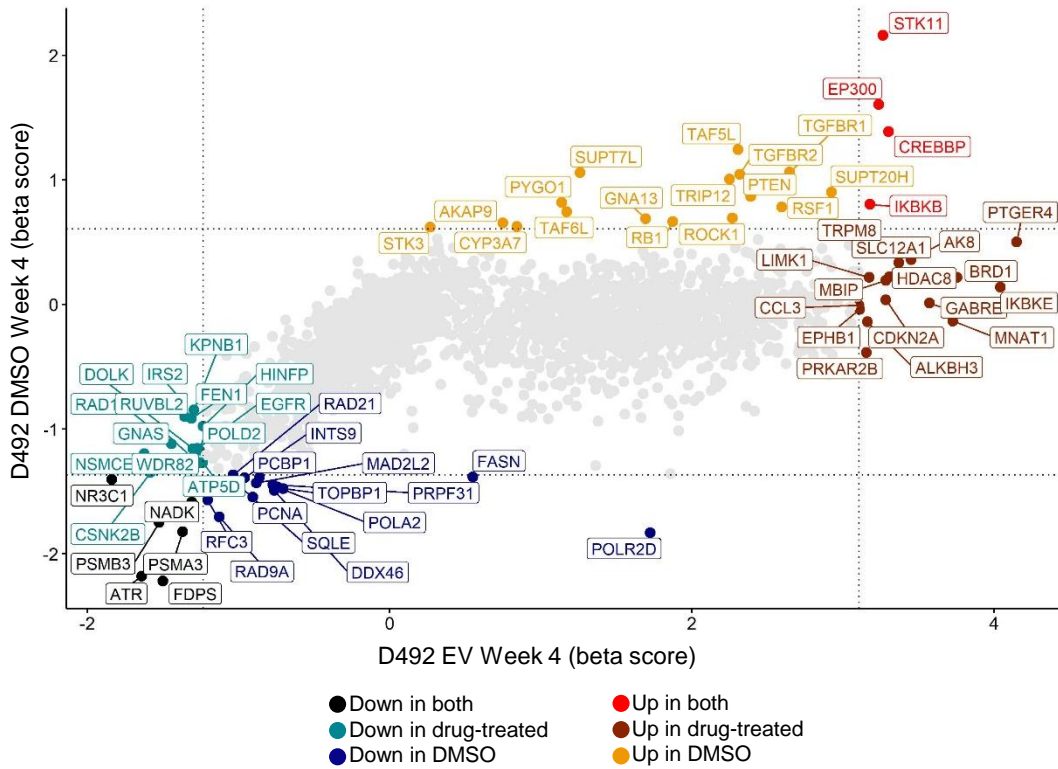

# B

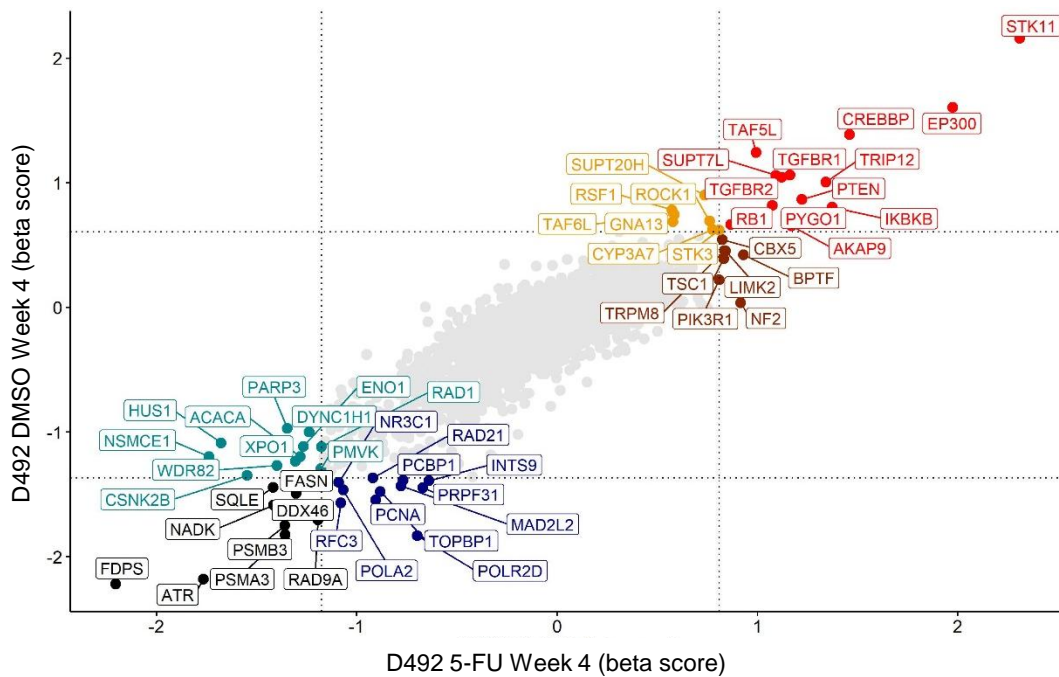

# Supplementary Figure S8 (cont)

C

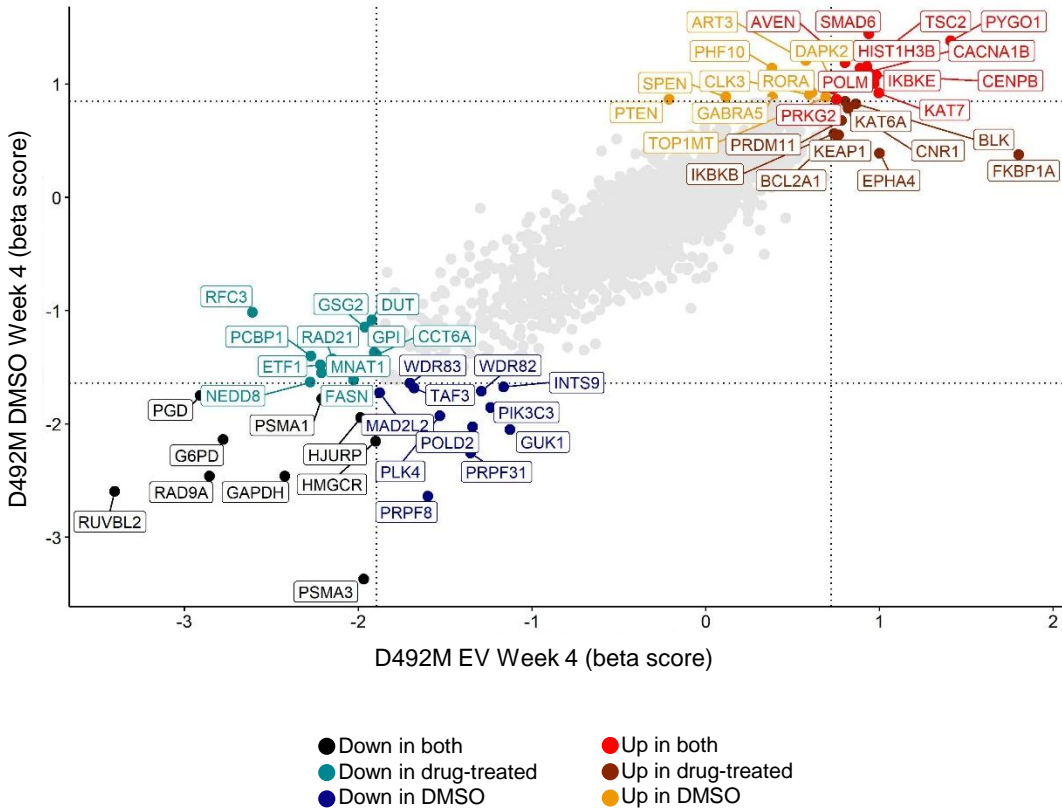

D

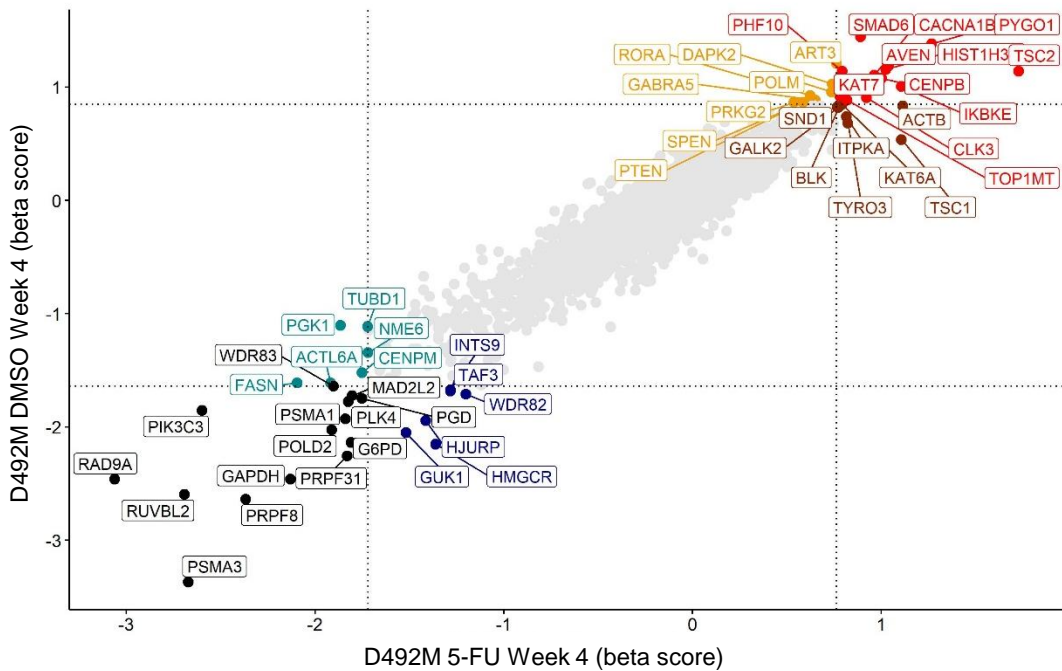

Supplement: Supplementary file 8 — Fig. S8. Phenotype‐specific gene cooperativity with everolimus and 5‐FU identified using MAGeCK‐MLE. CRISPR hits for everolimus (A, C) and 5‐FU (B, D) treated cells in indicated cell lines. Phenotype‐specific gene essentiality consists of genes with greater depletion in one cell line compared to the other. Shown is the beta score as calculated using MAGeCK‐MLE. The top 15 genes in each category are labeled. [file MOL2-15-2026-s013.pdf]

Supplementary Figure S9

Everolimus-treated

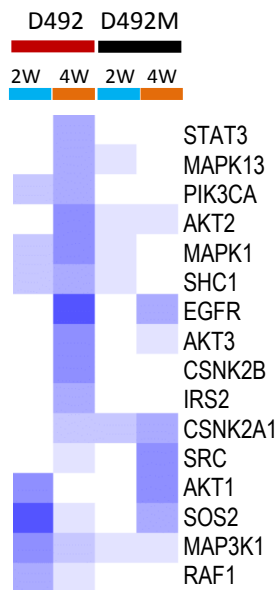

5-FU-treated

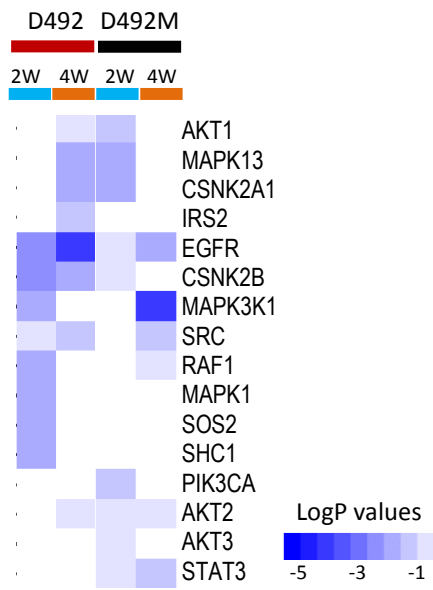

Supplement: Supplementary file 9 — Fig. S9. The significance of EGFR signaling‐related gene knockouts for toxicity in cells under ‘therapy pressure’. LogP values for EGFR signaling related genes (defined by Ingenuity Pathway Analysis) in everolimus‐ or 5‐FU‐ treated versus nontreated D492 or D492M cells at week two or week four. [file MOL2-15-2026-s014.pdf]

## Supplementary Figure S10

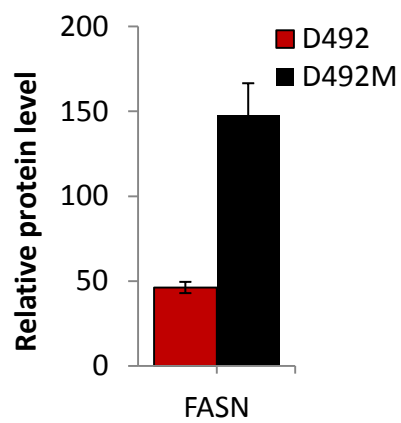

Supplement: Supplementary file 10 — Fig. S10. FASN protein expression in D492 and D492M cells. FASN expression level was detected by RPPA (average +/‐ StDev from three technical parallels). [file MOL2-15-2026-s001.pdf]

## Supplementary Figure S11

**A**

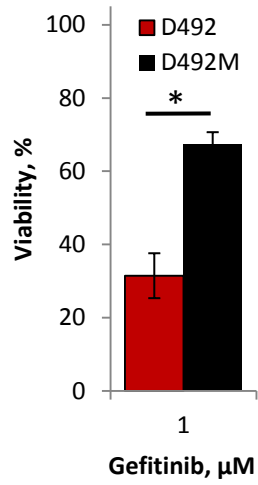

**B**

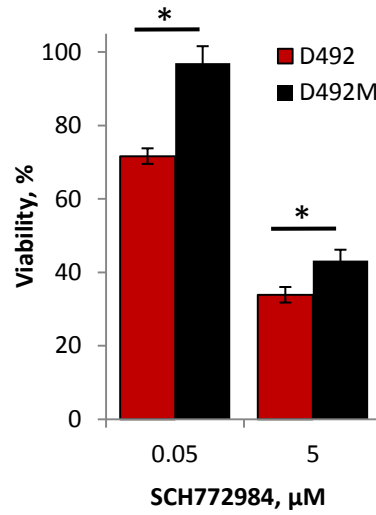

Supplement: Supplementary file 11 — Fig. S11. Sensitivity of 2D cultures of D492 and D492M to the EGFR and ERK inhibitors. Cells were grown as monolayers in 2D and treated for three days with the indicated concentrations of the EGFR inhibitor gefitinib (A) or the ERK inhibitor SCH772984 (B) before the cell survival was measured by the CTG method; average +/‐ SEM (n ≥ 4); *, p ≤ 0.05 by unpaired t‐test. [file MOL2-15-2026-s015.pdf]

# Supplementary Figure S12

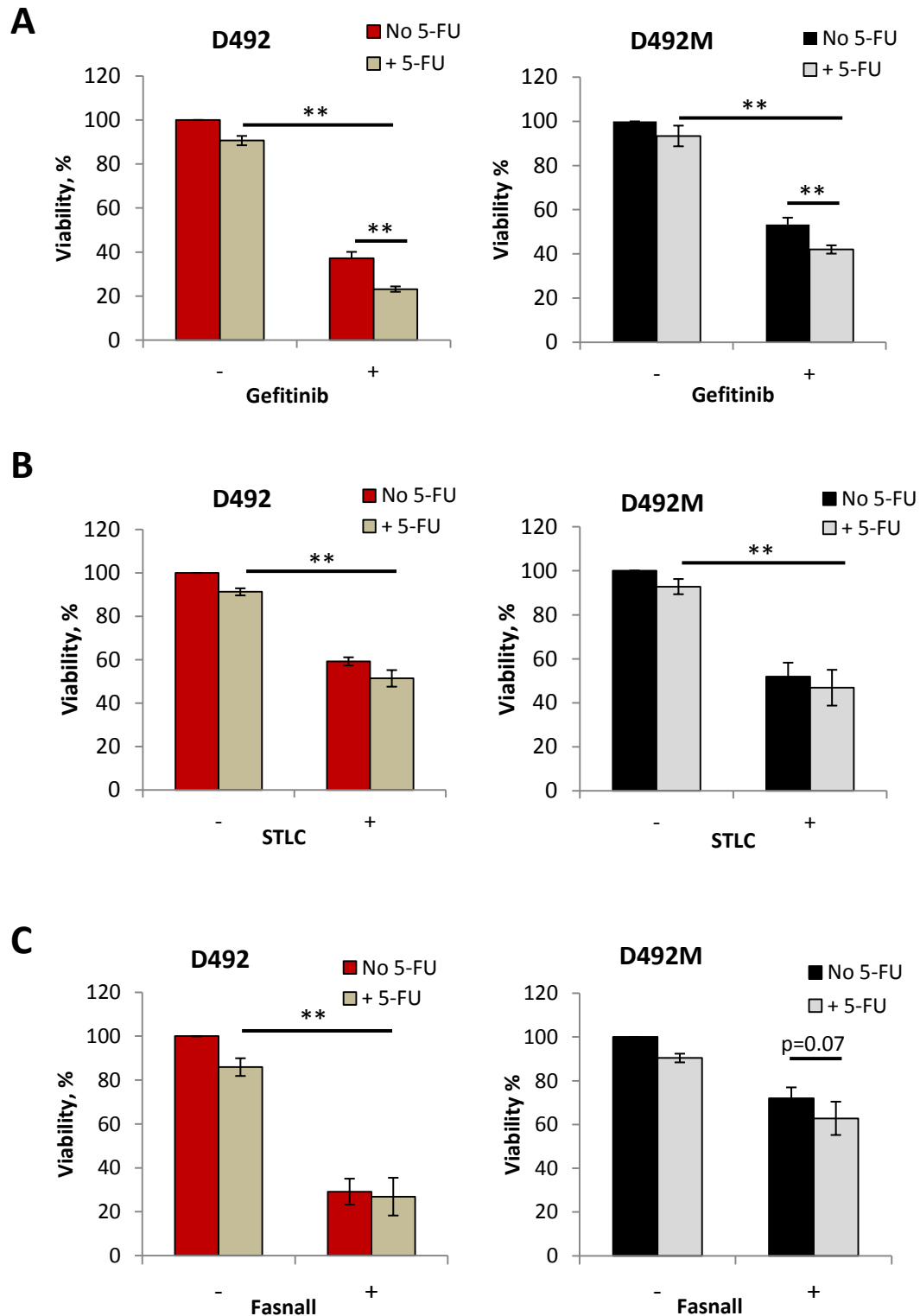

Supplement: Supplementary file 12 — Fig. S12. Sensitivity of D492 and D492M cells to gefitinib, STLC and Fasnall with/without additional treatment with 5‐FU. The cells were grown in 3D Matrigel and treated for eight days with 1 µM gefitinib (A), 5 µM (D492) or 1 µM (D492M) STLC (B) or 20 µM (D492) or 30 µM (D492M) Fasnall (C) in combination with 1 µM 5‐FU before the cell survival was measured by the CTG method; average +/‐ SEM (n = 3); ** p ≤ 0.05 by paired t‐test. [file MOL2-15-2026-s006.pdf]
