# Supplementary material for: Detection of phenotype‐specific therapeutic vulnerabilities in breast cells using a CRISPR loss‐of‐function screen
Source: Mol Oncol. 2021 May 1;15(8):2026–45. doi: 10.1002/1878-0261.12951 (PMC8333781; doi:10.1002/1878-0261.12951)

Supplementary Figure S2

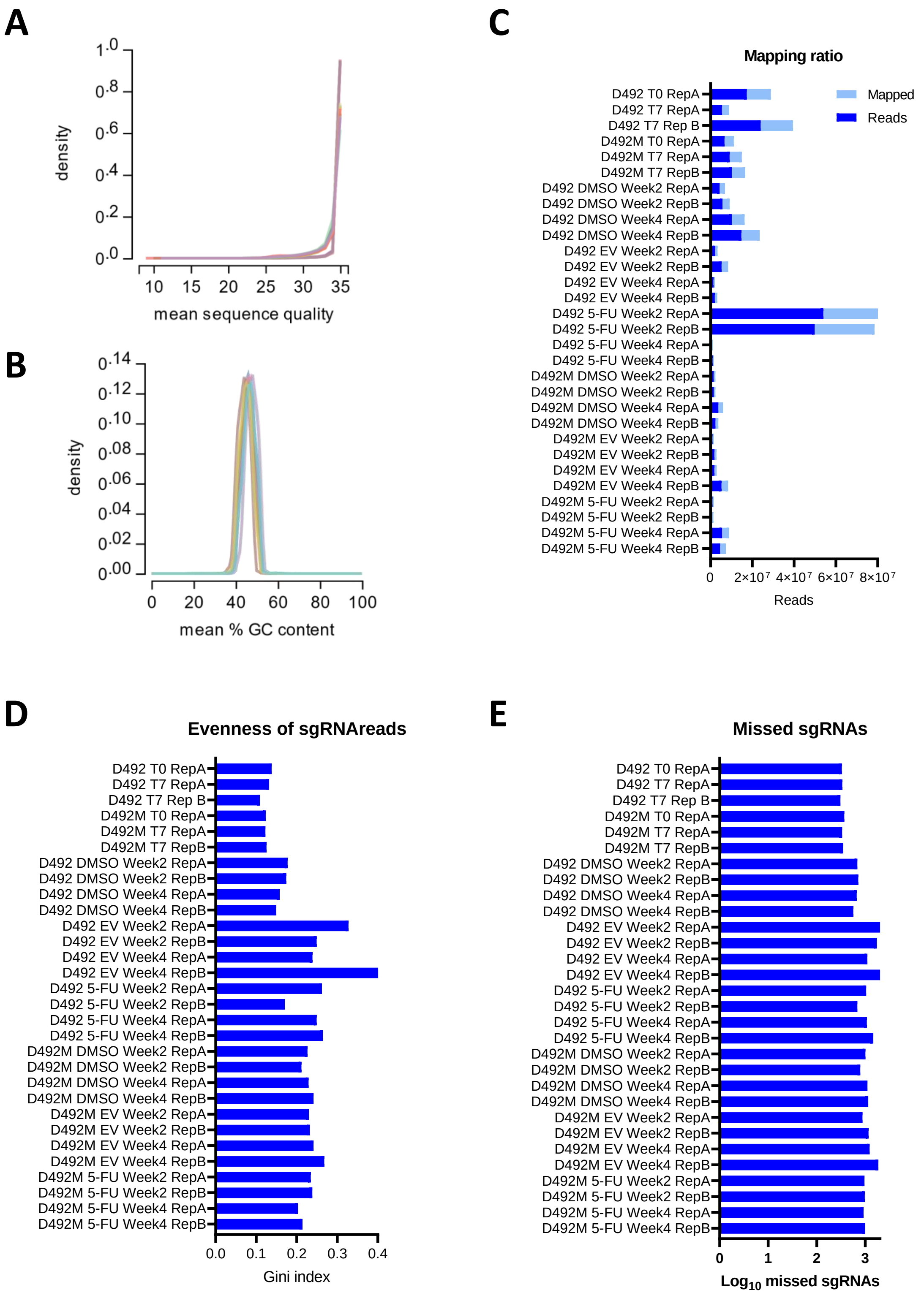

Supplementary Figure S2 (cont)

F

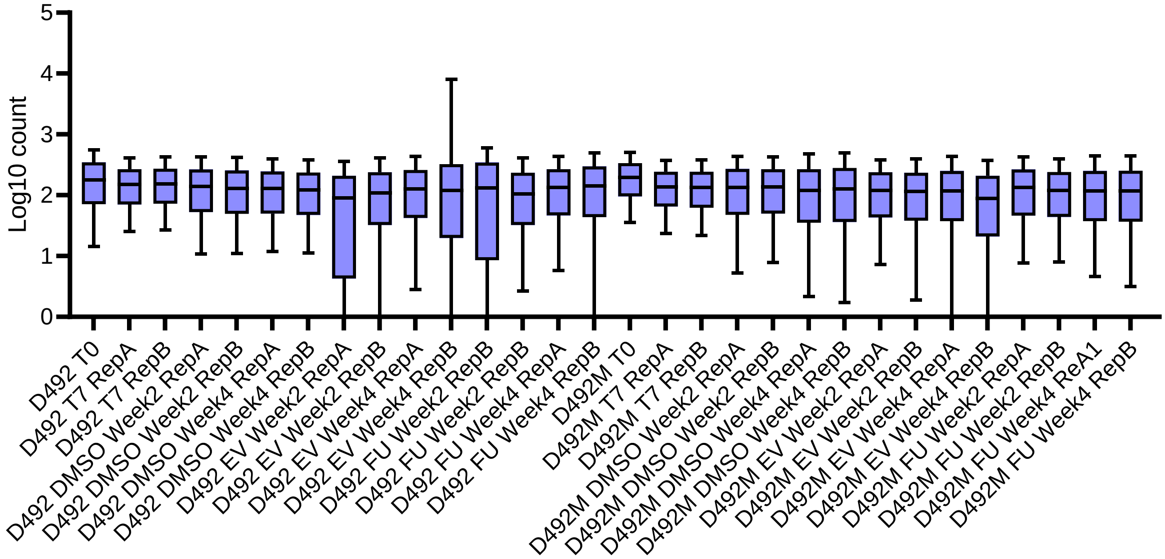

G

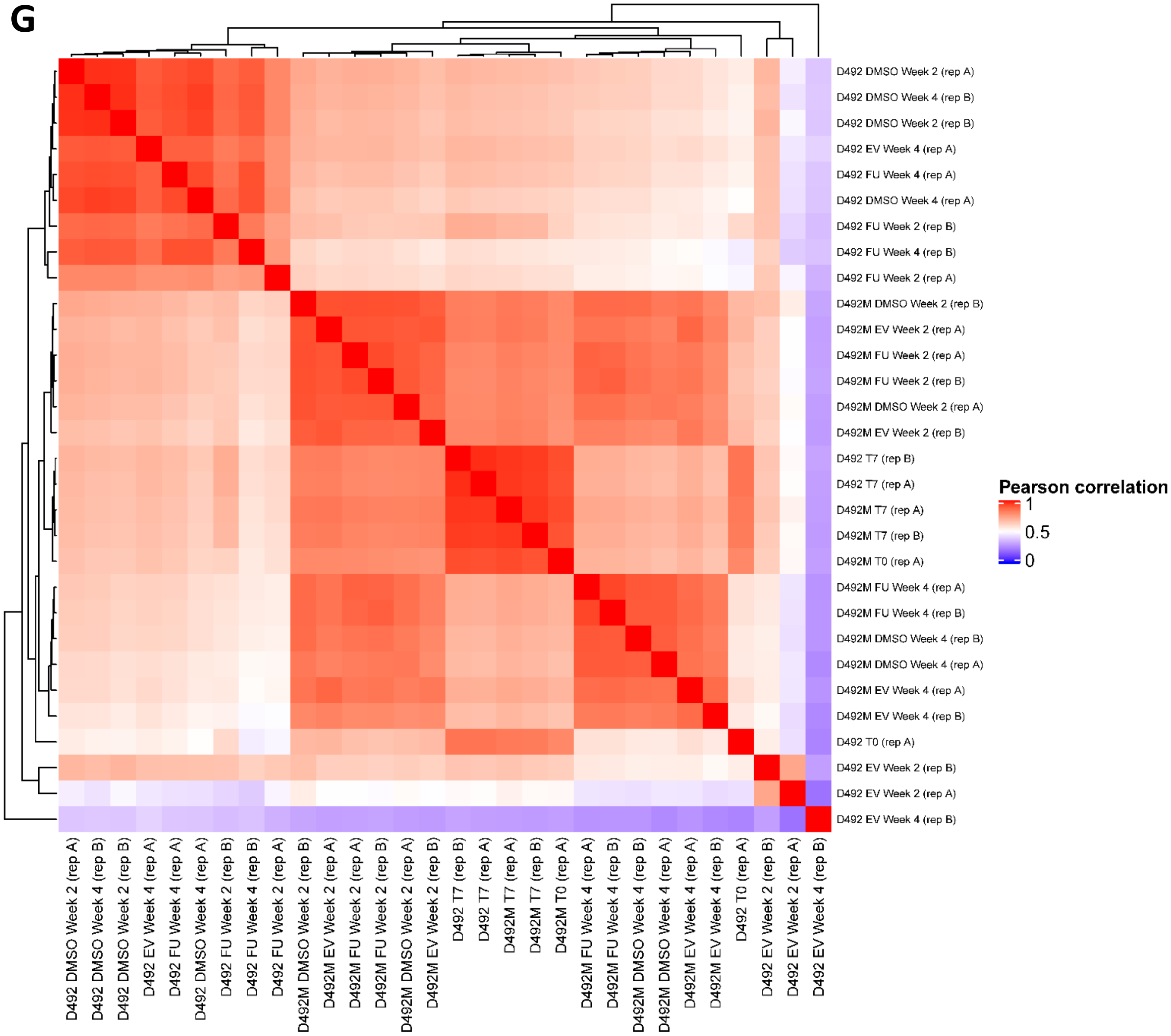

Supplement: Supplementary file 2 — Fig. S2. CRISPR screen sequencing and replicate quality analysis. A, Distribution of mean sequence quality. B, Distribution of GC content. C, Mapping ratio of all reads for indicated conditions. D, Gini index. E, Number of zero‐count sgRNAs per sample. F, normalized read count distribution, plotted as mean with 10‐90 percentile whiskers. G, Heat map of Pearson correlation scores for all CRISPR screen samples with unsupervised clustering. [file MOL2-15-2026-s009.pdf]
